# Comparison of automated white matter lesion segmentation approaches for use in large, multi-site data analyses in Parkinson’s disease

**DOI:** 10.3389/fnins.2026.1904361

**Published:** 2026-09-24

**Authors:** Sarah Al-Bachari, So Hoon Yoon, Phoebe Emson, Shauna Angell, John Cain, Aswin Abraham, Azeem Chughtai, Edward Sizer, Edwin Barnes, Maryam Al-Wardy, Siddarth Kannan, Rohan Paul-Thaper, Joanna Bright, Conor Owens-Walton, Corey T. McMillan, Johannes C. Klein, Ludovica Griffanti, Sophia I. Thomopoulos, Neda Jahanshad, Paul M. Thompson, Ysbrand D. van der Werf, Chris Vriend, Laura M. Parkes, Hedley C. A. Emsley, Anette Schrag, Hamied A. Haroon

**Affiliations:** 1 Department of Clinical and Movement Neurosciences, UCL Queen Square Institute of Neurology, University College London, London, England, United Kingdom; 2 Lancaster Medical School, Lancaster University, Lancaster, England, United Kingdom; 3 Department of Neurology, National Health Insurance Service Ilsan Hospital, Goyang, Republic of Korea; 4 Department of Neurology, Yonsei University College of Medicine, Seoul, Republic of Korea; 5 Department of Radiology, Royal Preston Hospital, Lancashire Teaching Hospitals, Preston, Lancashire, England, United Kingdom; 6 Faculty of Medical Sciences, University College London, London, England, United Kingdom; 7 School of Medicine, Faculty of Biology, Medicine and Health, University of Manchester, Manchester, England, United Kingdom; 8 Queen Elizabeth University Hospital, Glasgow, Scotland, United Kingdom; 9 Department of Behavioral Medicine, College of Medicine and Health Sciences, Sultan Qaboos University, Muscat, Oman; 10 Department of Neurosurgery, The Walton Centre, Liverpool, England, United Kingdom; 11 School of Medicine, University of Lancashire, Preston, England, United Kingdom; 12 Social, Genetic and Developmental Psychiatry Centre, King’s College London, London, England, United Kingdom; 13 Imaging Genetics Center, Mark and Mary Stevens Neuroimaging and Informatics Institute, Keck School of Medicine, University of Southern California, Marina del Rey, CA, United States; 14 Department of Neurology, Perelman School of Medicine, University of Pennsylvania, Philadelphia, PA, United States; 15 Division of Clinical Neurology, Nuffield Department of Clinical Neurosciences, Oxford Parkinson’s Disease Centre, University of Oxford, Oxford, England, United Kingdom; 16 Department of Psychiatry, Oxford Centre for Human Brain Activity, Centre for Integrative Neuroimaging (OxCIN), University of Oxford, Oxford, England, United Kingdom; 17 Department of Anatomy and Neurosciences, Amsterdam UMC, Vrije Universiteit Amsterdam, Amsterdam, Netherlands; 18 Compulsivity, Impulsivity and Attention Program, Amsterdam Neuroscience, Amsterdam, Netherlands; 19 Brain Imaging, Amsterdam Neuroscience, Amsterdam, Netherlands; 20 Department of Psychiatry, Amsterdam UMC, Vrije Universiteit Amsterdam, Amsterdam, Netherlands; 21 Faculty of Biology, Medicine and Health, School of Health Sciences, University of Manchester, Manchester, England, United Kingdom; 22 Geoffrey Jefferson Brain Research Centre, University of Manchester, Manchester, England, United Kingdom

**Keywords:** automated segmentation, magnetic resonance imaging, multi-site data, Parkinson’s disease, white matter lesions, white matter hyperintensities

## Abstract

**Background:**

The vascular role in Parkinson’s disease (PD) is emerging, yet the literature remains conflicted, motivating large-data analyses with greater statistical power. White matter lesions (WML) are an accepted imaging marker of small vessel disease. Accurate automated WML segmentation techniques are crucial for large-scale studies; however, evaluation of the optimum approach in PD is lacking. This study evaluated automated WML segmentation algorithms to determine the most accurate and reliable method, among those selected, for multi-site large data analysis in PD.

**Methods:**

We assessed whole-brain volumetric T1-weighted and FLAIR images from 201 PD patients (mean age, 66.6 ± 7.86 years) and 64 healthy controls (HC; mean age, 66.3 ± 8.67) across three datasets composed of different scanners, imaging parameters and lesion loads. WML were manually segmented to provide the gold standard, and four freely available automated algorithms were evaluated, FSL’s BIANCA, FreeSurfer, SPM’s LST-LPA and U-Net-pgs, using the performance metrics: Dice score, Hausdorff distance, recall, precision, F1 score, log absolute volume difference (LOGAVD) and intraclass correlation coefficient (ICC). Sub-analyses were performed based on lesion load, lobar regions and acquisition parameters.

**Results:**

U-Net-pgs produced the highest Dice score (PD: 0.46 ± 0.21; HC: 0.39 ± 0.21), recall (PD: 0.75 ± 0.24; HC: 0.58 ± 0.26), precision (PD: 0.48 ± 0.24; HC: 0.62 ± 0.25), F1 score (PD: 0.53 ± 0.21; HC: 0.54 ± 0.20) and ICC (PD: 0.87; HC: 0.89) and lowest Hausdorff distance (PD: 8.89 ± 3.96; HC: 6.33 ± 2.91) and LOGAVD (PD: 0.31 ± 0.31; HC 0.27 ± 0.30) across PD and HC. U-Net-pgs also showed overall superior performance in all lesion loads for PD and across brain regions in both PD and HC.

**Conclusion:**

Overall, of those we evaluated, U-Net-pgs emerged as the highest performing automated method across lesion loads and brain regions, for WML segmentation in PD and HC. The accuracy and reliability of U-Net-pgs, across various scanner and image acquisition parameters, make it a promising tool for large-scale analyses, facilitating future research investigating WML in PD.

## 1 Introduction

Parkinson’s disease (PD) is now recognized as the fastest-growing neurological disorder worldwide in terms of prevalence, disability and societal burden. About 6.1 million individuals globally had PD in 2016, and this number is anticipated to surpass 14 million by the year 2040 (Dorsey et al., 2018). PD is not only highly variable in terms of clinical presentation, with over 50 possible symptoms, but also in underlying neurobiology (Espay et al., 2017; Mestre et al., 2021). Within this diversity, there is an urgent need to identify groups who will benefit from specific treatments, i.e., a move towards a precision medicine approach (Thenganatt and Jankovic, 2014; Greenland et al., 2019; Marras et al., 2020; Hu et al., 2024). Due to the heterogeneity in PD, large-scale data analysis is required to enable subtyping and identify at-risk groups who may be responsive to certain treatments.

The vascular role in PD is rapidly emerging. The biological underpinnings lie at the level of the neurovascular unit, with blood-brain barrier dysfunction and cerebral hypoperfusion interacting with other key pathogenic processes that contribute to selective neuronal loss (Jellinger, 2003; Zlokovic, 2008; Al-Bachari et al., 2017; Sweeney et al., 2018; Al-Bachari et al., 2020). Large, multi-center longitudinal studies of PD have demonstrated cerebral vascular changes associated with greater PD-related disability and more severe gait, cognitive and mood phenotypes, that tend to be less responsive to levodopa treatment (Stojkovic et al., 2018; Chen et al., 2020; Lee et al., 2020). These vascular changes may not only accompany neurodegeneration but actively contribute to it, holding promise as a target for a disease-modifying treatment (Monti et al., 2022; Paul and Elabi, 2022; Kieliszek-Ryba et al., 2025), yet a vascular biomarker is lacking.

MRI markers of small vessel disease include white matter lesions (WML), lacunes, enlarged perivascular spaces, and cerebral microbleeds (Wardlaw et al., 2013; Chojdak-Łukasiewicz et al., 2021). Of these, WML are the most commonly measured marker and are particularly relevant in PD as they are associated with motor and cognitive deficits (Joutel and Chabriat, 2017; Duering et al., 2023). WML appear as hyperintensities on T2-weighted fluid-attenuated inversion recovery (FLAIR), while they appear as hypointensities on T1-weighted (T1w) MRI. Their measurement is non-invasive and highly reproducible, making WML an ideal neuroimaging marker for studies in PD. Several studies have demonstrated that higher WML burden correlates with worse outcomes in PD, including cognitive decline, gait disturbances, and increased risk of dementia (González-Redondo et al., 2012; Kandiah et al., 2013; Sunwoo et al., 2014; Compta et al., 2016; Malek et al., 2016; Ciliz et al., 2018; Stojkovic et al., 2018; Pozorski et al., 2019; Wu et al., 2023; Zou et al., 2023). However, despite over a decade of research, there are conflicting results, with some studies showing no association of WML with clinical features or disease severity (Dalaker et al., 2009a,b; Despotović et al., 2015; Lee and Lee, 2016; Hanning et al., 2019). This discrepancy is likely due to both the heterogeneity of PD pathophysiology and methodological differences in identifying WML. For example, certain methods may miss small deep WML or have neuroanatomical inaccuracies (Torres-Simon et al., 2024). This may have clinical relevance, as if small lesions in certain regions are consistently missed, their correlations with clinical features will also be missed. Therefore, WML identification approaches need both volumetric and neuroanatomical accuracy to allow all potential associations to be elicited.

Manual methods remain the current gold standard for WML segmentation; however, they are labor intensive, time consuming and show inter-rater variability. Thus, it is imperative to identify an automated algorithm for quantifying WML burden and location accurately for use in large-scale analyses (Hotz et al., 2022). Numerous automated WML segmentation algorithms have been proposed, including supervised, unsupervised, and semi-supervised methods depending on the quantity of expertly annotated data available. These methods have been extensively described elsewhere, yet in summary, each technique has its advantages and disadvantages, including poor performance in low lesion loads, requiring a high degree of human intervention and expertise, the requirement of a training set and varying false positive and false negative rates (Caligiuri et al., 2015). Few studies have compared the performance and validity of these different methods, particularly in heterogeneous datasets.

MRI studies are often small and fail to account for the heterogeneity of PD. Pooling data from multiple cohorts, often using consortium data, aims to overcome this challenge (Thompson et al., 2020). Consortium data have contributed significantly to our understanding of various disease processes (Weaver et al., 2021; Chen et al., 2025), yet certain challenges still prevail, namely differences in study protocols including scanner parameters, imaging acquisitions and clinical data collected (Zugman et al., 2022; Giehl et al., 2024). Therefore, imaging analysis methods that can withstand variations in scanner and image acquisition parameters need to be sought to allow for more uniform analysis in pooled datasets.

We here compared several freely available automated WML segmentation algorithms that have recently shown promise in producing accurate WML segmentations mostly in healthy aging and cognitively impaired populations (Heinen et al., 2019; Kuijf et al., 2019; Vanderbecq et al., 2020; Hotz et al., 2022; Gaubert et al., 2023; Torres-Simon et al., 2024). These are: FSL’s Brain Intensity AbNormality Classification Algorithm (BIANCA) (Griffanti et al., 2016), FreeSurfer (Fischl, 2012), SPM’s Lesion Segmentation Tool Lesion Prediction Algorithm (LST-LPA) (Schmidt et al., 2012) and UNet-pgs (Ronneberger et al., 2015; Li et al., 2018; Park et al., 2021). These approaches are summarized in Table 1. To the authors’ knowledge, these approaches have not been tested specifically to assess WML in PD, nor in PD consortium data that includes different scanners and variable imaging parameters such as slice thickness, voxel size and field strength, all of which may impact the accuracy of WML segmentations (Khademi et al., 2021; Tran, 2022).

**TABLE 1:** Summary of the automated white matter lesion (WML) segmentation approaches used in this study.

Name	Summary
BIANCA (Griffanti et al., 2016)	BIANCA is a supervised WML segmentation tool that applies a k-nearest neighbor classifier. It requires a training set of manually segmented lesion masks which are used to determine the similarity between features of the target voxel and those in the training set to estimate the lesion probability for each voxel. This results in a probabilistic map of WML; a threshold can be applied to binarize these.
FreeSurfer	FreeSurfer applies an automated pipeline to the T1w image including intensity normalization, brain extraction, surface reconstruction and volumetric labeling steps to delineate cortical and subcortical structures. A probabilistic anatomical atlas derived from manually annotated MRI datasets is used to assign each voxel to the most likely tissue class based on spatial context and intensity features. Results include a binary map of white matter hypointensities from the T1w image.
LST-LPA (LPA) (Schmidt et al., 2012)	LST-LPA uses a logistic-regression classifier trained on FLAIR images. A lesion belief map is generated from the training set and used to estimate the lesion probability at each voxel. The spatial information is combined with image intensity information to form a lesion map for each subject. This results in a probabilistic map of WML and a threshold can be applied to binarize these.
UNet-pgs (U-Net) (Li et al., 2018; Park et al., 2021)	UNet-pgs (referred to simply as U-Net in this paper) is a supervised convolutional neural network approach which can be applied to medical image segmentation. It is trained on co-registered T1w and FLAIR images using highlighting foregrounds to improve sensitivity for small or boundary lesions affected by partial volume effects. This results in a binary map of WML.

http://surfer.nmr.mgh.harvard.edu/; www.statistical-modelling.de/lst.html.

The aim of this study was to compare and identify an optimal automated WML segmentation approach for large multi-site data in PD which is robust to varying scanner and imaging parameters. The following questions were to be answered by this study:

1. Assessment of segmentation performance for use in large-scale multi-site data: *Which automated approach provides estimates that are most consistent with the gold standard of manual segmentations, assessed by established performance metrics* (Kuijf et al., 2019)*?*
2. Validations using visual rating scales and age: *How do the WML segmentations provided by the different approaches relate to Fazekas and Wahlund scores and chronological age?*

## 2 Materials and methods

### 2.1 Participants

Demographic and brain MRI data were gathered from three datasets: the Montreal Neurological Institute Biobank: Clinical Biological Imaging and Genetic Repository (C-BIG) (Das et al., 2022), the Parkinson’s Progression Markers Initiative (PPMI)^1^ and the University of Pennsylvania (UPenn).^2^ All participants underwent comprehensive clinical and imaging assessments. Written informed consent for participation and sharing of anonymized data was obtained from all participants at the relevant site. PD diagnosis was made based on the UK Parkinson’s Disease Brain Bank or Movement Disorder Society clinical criteria. Participants with any other significant CNS disorders were excluded. Healthy controls (HC) did not have PD or significant other CNS disorders.

### 2.2 MRI data acquisition

All participants underwent 1.5T or 3T 2D or 3D FLAIR and 3D T1w imaging. Scanner parameters including voxel sizes, repetition times, echo times and slice thickness varied (Table 2). All images were visually inspected for quality prior to analysis to confirm that no motion artifacts or poor image quality could substantially impact segmentations.

**TABLE 2:** Summary of MRI scanner parameters.

Parameter	Parkinson’s disease	Control
Sample size	201	64
Data source	C-BIG: 48 (23.9%); PPMI: 96 (47.8%); UPenn: 57 (28.4%)	C-BIG: 7 (10.9%); PPMI: 44 (68.8%); UPenn: 13 (20.3%)
Low WML load	C-BIG: 29 (20.0%); PPMI: 79 (54.5%); UPenn: 37 (25.5%)	C-BIG: 6 (10.5%); PPMI: 39 (68.4%); UPenn: 12 (21.1%)
Medium WML load	C-BIG: 11 (28.2%); PPMI: 14 (35.9%); UPenn: 14 (35.9%)	C-BIG: 0 (0.0%); PPMI: 3 (100.0%); UPenn: 0 (0.0%)
High WML load	C-BIG: 8 (47.1%); PPMI: 3 (17.6%); UPenn: 6 (35.3%)	C-BIG: 1 (25.0%); PPMI: 2 (50.0%); UPenn: 1 (25.0%)
MRI field strength	1.5T: 61 (30.3%); 3T: 140 (69.7%)	1.5T: 27 (42.2%); 3T: 37 (57.8%)
Low WML load	1.5T: 50 (34.5%); 3T: 95 (65.5%)	1.5T: 25 (43.9%); 3T: 32 (56.1%)
Medium WML load	1.5T: 9 (23.1%); 3T: 30 (76.9%)	1.5T: 1 (33.3%); 3T: 2 (66.7%)
High WML load	1.5T: 2 (11.8%); 3T: 15 (88.2%)	1.5T: 1 (25%); 3T: 3 (75%)
Scanner manufacturer	GE: 55 (27.4%); Philips: 18 (9%); Siemens: 128 (63.7%)	GE: 25 (39.1%); Philips: 11 (17.2%); Siemens: 28 (43.8%)
Low WML load	GE: 46 (31.7%); Philips: 15 (10.3%); Siemens: 84 (57.9%)	GE: 21 (36.8%); Philips: 10 (17.5%); Siemens: 26 (45.6%)
Medium WML load	GE: 8 (20.5%); Philips: 2 (5.1%); Siemens: 29 (74.4%)	GE: 2 (66.7%); Philips: 1 (33.3%); Siemens: 0 (0.0%)
High WML load	GE: 1 (5.9%); Philips: 1 (5.9%); Siemens: 15 (88.2%)	GE: 2 (50.0%); Philips: 0 (0.0%); Siemens: 2 (50.0%)
T1-weighted		
Acquisition type	3D: 201 (100%)	3D: 64 (100%)
Slice thickness (mm)	1.0–2.0	1.0–2.0
Voxel size (mm)	[0.43–1.51] × [0.43–1.25] × [0.72–4.00]	[0.43–2.00] × [0.43–1.25] × [0.49–2.00]
TE (s)	0.002–0.009	0.003–0.005
TR (s)	0.007–2.400	0.007–2.400
TI (s) (where applicable)	0.04–1.10	0.40–1.10
Flip angle (°)	8–90	8–20
Matrix size	[84–512] × [192–512] × [34–320]	[80–512] × [192–512] × [72–512]
FLAIR		
Acquisition type	2D: 144 (71.6%)/3D: 57 (28.4%)	2D: 51 (79.7%)/3D: 13 (20.3%)
Low WML load	2D: 108 (74.5%)/3D: 37 (25.5%)	2D: 45 (78.9%)/3D: 12 (21.1%)
Medium WML load	2D: 25 (64.1%)/3D: 14 (35.9%)	2D: 3 (100.0%)/3D: 0 (0.0%)
High WML load	2D: 11 (64.7%)/3D: 6 (35.3%)	2D: 3 (75.0%)/3D: 1 (25.0%)
Slice thickness (mm)	1.0–6.0	1.0–5.0
Voxel size (mm)	[0.45–1.05] × [0.43–1.05] × [1.00–7.47]	[0.43–1.00] × [0.43–1.05] × [1.00–8.47]
TE (s)	0.074–0.292	0.100–0.292
TR (s)	6.00–11.00	6.00–12.65
TI (s)	1.65–2.80	2.00–2.80
Flip angle (°)	90–180	90–180
Matrix size	[160–576] × [256–576] × [20–256]	[160–560] × [256–560] × [18–256]

Data presented for the entire Parkinson’s disease and control groups and lesion load subgroups where applicable. TE, Echo Time; TR, Repetition Time; TI, Inversion Time; 2D, Two-dimensional; 3D, Three-dimensional. The range of the z-dimension for voxel sixe reflects the space between slices (i.e., slice thickness + gap).

### 2.3 Manual segmentation of WML

WML were manually segmented to establish a gold standard for assessing the performance of WML segmentation by automated methods. FLAIR images for each participant were manually segmented by two trained raters (PE and SA-B) in the axial plane using ITK-SNAP software. PE and SA-B were both trained by consultant neuroradiologist JC. All manual segmentations were performed blinded to the automated segmentations.

### 2.4 Automated WML segmentation algorithms

The following automated approaches were applied to the complete dataset: BIANCA, FreeSurfer, LPA and U-Net. To allow fair comparison of the best performance of each algorithm, all approaches were optimized to the best of our ability with specific changes outlined below. For those algorithms for which threshold selection was required, threshold optimization and performance evaluation was performed in a subset of subjects from all three datasets so there was no bias to one particular dataset. All segmentation scripts are openly available (Haroon, 2026a).

#### 2.4.1 BIANCA

Both T1w and FLAIR images were used as inputs to maximize classifier performance. Bias field correction was applied to the images and the T1w image registered to the FLAIR image using the FSL tools FMRIB’s Automated Segmentation Tool (FAST) and the Linear Image Registration Tool, respectively (Zhang et al., 2001; Jenkinson et al., 2012).

Two training strategies were applied. A leave-one-out approach (BIANCA-LOO) segmented each subject’s image using all remaining subjects as the reference set. This was done for both PD and HC separately and required all data to be manually labeled. As this approach would not be practical for unseen multi-site data, a trained-model approach (BIANCA-training), which generated training data from two of the three datasets then applied to the third dataset, was also evaluated. Due to the low number of HC, this was only done in PD.

Binary lesion masks were generated using thresholds of 0.9 and 0.99 (BIANCA (0.9), BIANCA (0.99)), based on in-house threshold testing, visual inspection of the outputs and recommended optimal thresholds (Griffanti et al., 2016).

#### 2.4.2 FreeSurfer

T1w images were used as the inputs for FreeSurfer (version 7.2.0). To optimize the approach, bias field correction using FMRIB’s Automated Segmentation Tool (FAST) was applied (Zhang et al., 2001). Both bias field corrected (FreeSurfer-FAST) and uncorrected (FreeSurfer) approaches were used in the study. FreeSurfer’s tissue segmentation and parcellation pipeline was used with default settings to identify white matter hypointensities from T1w images which were assumed to reflect WML.

The outputs in T1w space were transformed to FLAIR space using FSL (Jenkinson et al., 2012) to allow comparisons with the manual segmentations in FLAIR space. The co-registered masks were visually inspected to ensure successful co-registration and ensure spatial agreement between manual and automated segmentations.

#### 2.4.3 LPA

Both T1w and FLAIR images were used as inputs to maximize classifier performance [version 3.0.0 of the LST toolbox^3^ for SPM12 in MATLAB (R2025a, The Mathworks)]. Bias field correction and transformation of the T1w image to FLAIR space as described above were also applied. Both bias field corrected (LPA-FAST) and uncorrected (LPA) approaches were used in the study.

To generate binary lesion masks, thresholds of 0.4 and 0.5 [LPA (0.4), LPA (0.5)] were applied to the probability maps based on in-house threshold testing, visual inspection of the outputs and previous studies (Torres-Simon et al., 2024).

#### 2.4.4 U-Net

We adapted a pre-processing step to register the FLAIR to the T1w rather than vice versa due to T1w images often demonstrating higher anatomical resolution and contrast. The outputs in T1w space were transformed to FLAIR space using FSL (Jenkinson et al., 2012) as above.

### 2.5 Grouping according to WML loads

The WML load was determined from the volume of the manual segmentations using ITK-SNAP (Yushkevich et al., 2006). All participants were divided into three groups based on the load of WML. Participants with WML loads less than 5 cm^3^ were grouped into the “low WML load group,” those with more than 15 cm^3^ were grouped into the “high WML load group” and the rest were grouped into the “medium WML load group” (Griffanti et al., 2016; de Sitter et al., 2017; Ong et al., 2022). This classification was based on the total lesion volume regardless of whether the WML were contiguous or sparse. The performance of each automated WML approach was analyzed using the performance metrics below according to these subgroups in PD and the low WML load group in HC.

### 2.6 Performance metrics

All performance metrics were computed in MATLAB (R2025a, The Mathworks) using in-house code which is openly available for further use (Haroon, 2026b). The metrics used are summarized in Table 3. This combination of metrics was selected to not only ensure volumetric and spatial agreement between the automated approaches and manual segmentation, but to understand the influence of true/false positives/negatives.

**TABLE 3:** Summary of the metrics used to assess the performance of the automated white matter lesion (WML) segmentation approaches.

Performance metric	Equation	Explanation
Dice similarity coefficient (Dice score)	Dice score = (2 × Intersection)/(Volume of Set A + Volume of Set B)	Assesses similarity and overlap between two sets, A and B, by measuring how closely the predicted region spatially matches the ground truth region. Scored from 0 to 1, 1 signifying an exact match between the predicted segmentation and the ground truth. Dice score has a bias with larger WML volumes giving higher Dice scores.
Hausdorff distance	H (A, B) = max [*sup inf* d(a,b), *sup inf* d(b,a)] d(a,b): distance between points a and b *sup*: maximum value in a set *inf*: minimum value in a set	Quantifies the maximum boundary mismatch between two sets by measuring the largest distance from a point in one set (A) to the nearest point in the other set (B). The smaller the distance, the closer two sets are to each other. Particularly sensitive to outliers as it considers the maximum deviation. A lower Hausdorff distance is associated with better performance.
Recall (sensitivity)	Recall = True Positives/(True Positives + False Negatives)	Quantifies the proportion of true lesions that are correctly identified. High recall indicates that the majority of true lesions were successfully detected, whereas low recall suggests under-segmentation. Distinct lesions were identified by using Matlab’s *bwlabeln* function with a 3D voxel connectivity of 26 (R2025a, The Mathworks).
Precision (positive predictive value)	Precision = True Positives/(True Positives + False Positives)	Assesses the proportion of predicted lesions that are true lesions. High precision indicates that the model produces few false positive detections, whereas low precision suggests over-segmentation. Distinct lesions were identified by using Matlab’s *bwlabeln* function with a 3D voxel connectivity of 26 (R2025a, The Mathworks).
F1 score	F1 score = (2 × True Positives)/[(2 × True Positives) + False Positives + False Negatives]	A composite metric that represents the harmonic mean of recall and precision, providing a single measure that balances false negatives and false positives. A high F1 score indicates a favorable balance between lesion detection sensitivity and prediction reliability.
Log-transformed absolute volume difference (LOGAVD)	LOGAVD = ln(Vseg + 1) - ln(Vgt + 1) Vseg: volume of the segmented region Vgt: volume of the ground truth region	Quantifies the difference in volume between the model segmentation object and the ground truth object. The two volumes are compared on a natural-logarithmic scale, which limits the influence of the largest lesion loads; the offset of 1 mL keeps the metric finite when either volume is zero. A smaller LOGAVD indicates that the segmented volume closely matches the ground truth volume, whereas a larger LOGAVD suggests a discrepancy between the segmented and ground truth volumes, indicating that the model may over- or under-segment.
Intraclass correlation coefficient (ICC)	ICC(A,1) = (MS_*R*_ - MS_*E*_)/{MS_*R*_ + (k-1)MS_*E*_ + k(MS_*C*_-MS_*E*_)/n} MS_*R*_: mean square for rows (subjects) MS_*C*_: mean square for columns (methods) MS_*E*_: error mean square k: number of methods n: number of subjects	Quantifies the absolute agreement between volumes obtained from manual segmentation and those derived from automated approaches. Scored from 0 to 1, 1 signifying perfect agreement between the automated segmentation volume and the manual segmentation volume.

### 2.7 Effect of scanner and imaging parameters

Our study uses multi-site MRI data with varying scanner and imaging parameters. In practice, data harmonization would be performed prior to analysis; however, this study aimed to identify an optimal automated WML segmentation algorithm which is robust to these varying parameters and heterogeneous data. Nonetheless, to assess the impact of specific scanner and imaging parameters on the performance of the automated approaches, we performed sub-analyses to assess the effect of data source (C-BIG; PPMI; UPenn), field strength, (1.5T; 3T) FLAIR image dimension (2D; 3D) and MRI scanner manufacturer (GE; Philips; Siemens) using the performance metrics outlined above.

### 2.8 Lobar subgroup analysis

We investigated whether the performance of each automated WML approach differed according to the WML location. For this, we measured WML distribution using predefined lobes: frontal, temporal, parietal, occipital, cingulate, insular lobe, and remaining unparcellated white matter corresponding with the centrum semiovale and corpus callosum categorized as deep white matter (DWM). Each lobe was parcellated using cortical-adjacent white matter parcellation by modifying the cortical parcellation provided by FreeSurfer (Desikan et al., 2006). Dice and LOGAVD were calculated in each lobe in both PD and HC to statistically compare spatial and volumetric agreement with the gold standard manual segmentation between approaches.

### 2.9 Correlation with age and visual rating scales

We also investigated the correlation of WML measures with visual rating scales and age. Visual rating scales are used clinically to identify the location and severity of WML, albeit they do not quantify WML volume. The Fazekas and Wahlund scales are most commonly used for evaluating WML burden in clinical practice and trials and are therefore a clinically meaningful comparator for assessing the performance of automated approaches (Fazekas et al., 1987; Wahlund et al., 2001). In addition, age is one of the most important risk factors for WML, and it is well established that WML volume increases with age (regardless of other co-morbidities), so a positive correlation between age and WML burden would be expected (Griffanti et al., 2018; Hotz et al., 2022). This analysis was only performed within the PD group, as the majority of HC subjects were within the low lesion load group. PE conducted the visual ratings.

### 2.10 Visual assessment of WML segmentations

In addition to quantitative metrics, all outputs from each of the automated approaches were visually inspected in ITK-SNAP (by SA-B). This was done to better understand the performance of the approaches through review of the outputs, aiming to detect any systematic errors in the approaches we used and identify any patterns in under- or over-segmentation or missing lesions.

### 2.11 Statistical analysis

All statistical analyses were performed using R software, version 4.3.3 (R Foundation for Statistical Computing, R-project.org). Linear mixed-effects models were fitted with the *lme4* package, version 1.1-35.1 (Bates et al., 2015). Estimated marginal means, their 95% confidence intervals, and the Tukey-adjusted pairwise contrasts were obtained with the *emmeans* package, version 1.10.0 (Lenth, 2016); Kenward–Roger denominator degrees of freedom were computed through the *pbkrtest* package, version 0.5.2 (Halekoh and Højsgaard, 2014). Intraclass correlation coefficients and their confidence intervals were computed in base R following McGraw and Wong (McGraw and Wong, 1996). A *p* < 0.05 was considered statistically significant.

#### 2.11.1 Inter-rater reliability of manual segmentations

Inter-rater reliability was assessed using Dice score and ICC on a subset of thirty scans, comprising ten scans from each of the low, medium and high WML load groups, to ensure adequate agreement between the two raters.

#### 2.11.2 Manual vs. automated segmentations

Demographic features, clinical characteristics, and MRI scanner parameters were compared using independent *t*-tests and Pearson’s χ^2^-tests depending on the distribution of the data. Six evaluation metrics: Dice, Hausdorff, LOGAVD, recall, precision and F1 score were used to compare the manual segmentation with the output from each automated approach. Each participant contributed one segmentation per automated approach, so the six evaluation metrics constitute repeated measures nested within participants. We therefore analyzed each metric with a linear mixed-effects model containing automated approach as a fixed effect and subject as a random intercept, using the model metric ∼ automated approach + (1 | participant). Models were fitted by restricted maximum likelihood; separate models were fitted in the PD and control cohorts and within each lesion-load group. A test of whether performance differed across approaches was obtained from a likelihood-ratio test comparing the full model with an intercept-only model containing the same random intercept, both refitted by maximum likelihood. Results are reported as the model-estimated marginal mean with 95% confidence intervals. *Post hoc* comparisons were performed by pairwise contrasts between automated approaches, with *p*-values adjusted by the Tukey method (Tukey, 1949) and denominator degrees of freedom obtained by the Kenward–Roger approximation (Kenward and Roger, 1997). Agreement between manual and automated lesion volumes was quantified with the ICC in a two-way mixed-effects model with a single measurement and an absolute-agreement definition [ICC(A,1)], computed on log(x + 1)-transformed volumes with 95% confidence intervals, which is appropriate when the approaches are treated as fixed effects and the objective is to evaluate absolute agreement with the manual reference rather than consistency alone and account for systematic over- or under-estimation by an approach.

#### 2.11.3 Correlation with age and visual rating scales

Bivariate correlation analysis was performed to assess correlation between age and WML volumes from the manual segmentations and automated approaches. Partial correlation analysis after adjusting for age was used to form correlations with the visual rating scales Fazekas and Wahlund. Family-wise error rate corrected *p*-value of < 0.05 using Bonferroni correction was applied for multiple comparisons.

## 3 Results

### 3.1 Demographic and clinical characteristics of participants

The demographic and clinical features of the PD and HC groups are summarized in Table 4. Data were collected from 204 PD and 72 HC subjects; after exclusion of 3 PD and 8 HC participants due to failure of at least one automated approach to run, 201 PD and 64 HC subjects were included in the analysis. In the PD group, the mean age was 66.6 ± 7.86 years with 34.8% female. In the HC group, the mean age was 66.3 ± 8.67 with 43.8% female. Both age and sex did not significantly differ between the two groups. In the PD group, the mean Movement Disorder Society Unified Parkinson’s Disease Rating Scale (MDS-UPDRS) total score was 34.3 ± 14.74 and the Hoehn and Yahr stage was 1.92 ± 0.68. The mean Montreal Cognitive Assessment (MoCA) score significantly differed between the two groups: in the PD group, the average score was slightly lower, with 26.3 ± 3.30, compared to 28.1 ± 1.17 in the HC group. However, both means were within the normal cognitive range. The purpose of this study was not to study clinical correlations with WML, but to identify an optimal automated segmentation approach; however, detailed demographic features are provided in Table 4 to highlight disease variability. All scanner parameters are outlined in Table 2.

**TABLE 4:** Demographic features, clinical characteristics, and scanner parameters for participants.

Characteristic	Parkinson’s disease	Control	*p*-value
Number	201	64	–
WML load			**0.005**
Low WML load (%)	145 (72.1)	57 (89.1)	
Medium WML load (%)	39 (19.4)	3 (4.7)	
High WML load (%)	17 (8.5)	4 (6.2)	
Age, yr	66.6 ± 7.86 (*n* = 201)	66.3 ± 8.67 (*n* = 64)	0.802
Low WML load	64.9 ± 7.56 (*n* = 145)	66.2 ± 8.82 (*n* = 57)	0.321
Medium WML load	69.4 ± 6.71 (*n* = 39)	65.5 ± 7.03 (*n* = 3)	0.442
High WML load	74.3 ± 6.48 (*n* = 17)	67.3 ± 9.42 (*n* = 4)	0.089
Sex, female (%)	70 (34.8) (*n* = 201)	28 (43.8) (*n* = 64)	0.255
Low WML load	48 (33.1) (*n* = 145)	24 (42.1) (*n* = 57)	0.229
Medium WML load	16 (41.0) (*n* = 39)	1 (33.3) (*n* = 3)	1.000
High WML load	6 (35.3) (*n* = 17)	3 (75) (*n* = 4)	0.272
MDS-UPDRS total score	34.3 ± 14.74 (*n* = 135)	–	–
Low WML load	33.4 ± 14.32 (*n* = 104)	–	–
Medium WML load	36.7 ± 13.43 (*n* = 22)	–	–
High WML load	39.4 ± 21.65 (*n* = 9)	–	–
Hoehn and Yahr stage	1.92 ± 0.68 (*n* = 135)	–	–
Low WML load	1.87 ± 0.68 (*n* = 104)	–	–
Medium WML load	1.95 ± 0.58 (*n* = 22)	–	–
High WML load	2.44 ± 0.73 (*n* = 9)	–	–
MoCA total score	26.3 ± 3.30 (*n* = 135)	28.1 ± 1.17 (*n* = 44)	**< 0.001**
Low WML load	26.5 ± 3.27 (*n* = 104)	28.2 ± 1.17 (*n* = 39)	**< 0.001**
Medium WML load	26.3 ± 2.91 (*n* = 22)	27.0 ± 0.00 (*n* = 3)	0.255
High WML load	24.7 ± 4.36 (*n* = 9)	27.0 ± 0.00 (*n* = 2)	0.147

Values are expressed as mean ± standard deviation or number (percentage). P-values are the results of independent t-tests and Chi-squared tests, as appropriate. MDS-UPDRS, The Movement Disorder Society-Unified Parkinson’s Disease Rating Scale; MoCA, Montreal Cognitive Assessment; PD, Parkinson’s disease; WML, white matter lesions. Statistically significant values are represented in bold.

In the PD group, 72.1% had low lesion load, 19.4% had medium lesion load, and 8.5% had high lesion load. The proportion of low, medium, and high lesion load in the HC group was 89.1, 4.7, and 6.2% respectively. As most automated approaches perform better in higher lesion loads (Gaubert et al., 2023), a predominance of low lesion load was selected based on visual inspection of the FLAIR scans.

### 3.2 Inter-rater reliability of manual segmentation

Of the thirty manual segmentation samples, the median WML volume was 7.69 (IQR 14.70) cm^3^, with ten subjects in each of the low, medium and high lesion load groups. Median volumes for each of the groups were 1.39 (1.82) cm^3^, 7.68 (2.35) cm^3^, and 24.58 (11.41) cm^3^, respectively.

Adequate agreement between the two raters was confirmed by an ICC of 0.992 [95% CI 0.984–0.996] with *p* < 0.001 and a mean Dice score of 0.816 (SD 0.181).

### 3.3 Comparison of performance metrics among automated approaches

The results for the performance metrics in the PD and HC groups are summarized in Table 5 and Figures 1, 2. In PD, U-Net achieved the highest Dice score (0.46 ± 0.21 [0.44–0.49]), recall (0.75 ± 0.24 [0.71–0.78]), precision (0.48 ± 0.24 [0.45–0.51]), F1 score (0.53 ± 0.21 [0.51–0.56]) and ICC (0.87 [0.83–0.90]). *Post-hoc* analyses showed all metrics were statistically significantly higher than all other automated approaches. In HC, U-Net also achieved the highest Dice score (0.39 ± 0.21 [0.35–0.44]), recall (0.58 ± 0.26 [0.52–0.64]), precision (0.62 ± 0.25 [0.56–0.67]), F1 score (0.54 ± 0.20 [0.50–0.58]) and ICC (0.89 [0.82–0.93]). *Post-hoc* analyses showed all metrics were statistically significantly higher than all other automated approaches, except for the Dice score and recall with BIANCA-LOO (0.9).

**TABLE 5:** Performance metrics across the automated approaches in the PD and control group.

Performance metric	BIANCA-LOO (0.9)	BIANCA-LOO (0.99)	BIANCA-training (0.9)	BIANCA-training (0.99)	FreeSurfer	FreeSurfer-FAST	LPA (0.4)	LPA (0.5)	LPA-FAST (0.4)	LPA-FAST (0.5)	U-Net	*p*-value
PD												
Dice	0.42 ± 0.20* [0.39–0.45]	0.28 ± 0.16* [0.25–0.30]	0.39 ± 0.20* [0.36–0.41]	0.27 ± 0.17* [0.25–0.30]	0.21 ± 0.16* [0.18–0.24]	0.22 ± 0.17* [0.19–0.25]	0.29 ± 0.23* [0.26–0.32]	0.28 ± 0.23* [0.25–0.30]	0.29 ± 0.22* [0.27–0.32]	0.27 ± 0.21* [0.25–0.30]	**0.46 ± 0.21^†^ [0.44–0.49]**	< 0.001
Hausdorff	9.09 ± 4.65 [8.39–9.79]	9.07 ± 5.63 [8.34–9.75]	9.41 ± 4.83 [8.71–10.11]	9.14 ± 5.76 [8.44–9.84]	10.05 ± 5.30* [9.35–10.75]	10.01 ± 5.23* [9.31–10.71]	9.83 ± 4.61* [9.13–10.53]	9.36 ± 4.74 [8.66–10.06]	9.67 ± 5.08* [8.97–10.37]	9.35 ± 5.37 [8.65–10.05]	**8.89 ± 3.96^†^ [8.19–9.59]**	< 0.001
Recall	0.64 ± 0.24 * [0.60–0.67]	0.47 ± 0.25* [0.43–0.50]	0.65 ± 0.24* [0.62–0.69]	0.47 ± 0.23* [0.44–0.51]	0.27 ± 0.21* [0.24–0.30]	0.27 ± 0.22* [0.24–0.31]	0.36 ± 0.29* [0.33–0.39]	0.31 ± 0.27* [0.27–0.34]	0.39 ± 0.28* [0.36–0.42]	0.32 ± 0.26* [0.29–0.36]	**0.75 ± 0.24^†^ [0.71–0.78]**	< 0.001
Precision	0.27 ± 0.20* [0.24–0.30]	0.36 ± 0.24* [0.34–0.39]	0.17 ± 0.16* [0.14–0.20]	0.26 ± 0.20* [0.23–0.28]	0.09 ± 0.07* [0.07–0.12]	0.09 ± 0.07* [0.06–0.12]	0.25 ± 0.21* [0.22–0.28]	0.29 ± 0.23* [0.26–0.32]	0.29 ± 0.21* [0.26–0.32]	0.34 ± 0.24* [0.31–0.37]	**0.48 ± 0.24^†^ [0.45–0.51]**	< 0.001
F1 score	0.33 ± 0.19* [0.31–0.35]	0.35 ± 0.19* [0.33–0.37]	0.23 ± 0.16* [0.21–0.26]	0.28 ± 0.16* [0.26–0.30]	0.12 ± 0.07* [0.09–0.14]	0.12 ± 0.07* [0.09–0.14]	0.24 ± 0.16* [0.22–0.26]	0.24 ± 0.17* [0.21–0.26]	0.27 ± 0.16* [0.25–0.29]	0.26 ± 0.17* [0.24–0.28]	**0.53 ± 0.21^†^ [0.51–0.56]**	< 0.001
LOGAVD	0.34 ± 0.28 [0.29–0.39]	0.62 ± 0.41* [0.57–0.67]	0.37 ± 0.31 [0.32–0.42]	0.58 ± 0.42* [0.53–0.63]	0.54 ± 0.48* [0.49–0.59]	0.53 ± 0.50* [0.48–0.59]	0.41 ± 0.34 [0.36–0.46]	0.37 ± 0.33 [0.32–0.43]	0.42 ± 0.34* [0.37–0.47]	0.43 ± 0.34* [0.38–0.48]	**0.31 ± 0.31^†^ [0.26–0.37]**	< 0.001
ICC (A, 1)												
Coefficient	0.87 [0.83–0.90]	0.67 [-0.04 to 0.87]	0.84 [0.79–0.87]	0.68 [0.06–0.86]	0.62 [0.52–0.69]	0.61 [0.52–0.69]	0.83 [0.78–0.88]	0.85 [0.81–0.89]	0.82 [0.77–0.86]	0.81 [0.73–0.87]	**0.87^†^ [0.83–0.90]**	
*p*-value	< 0.001	< 0.001	< 0.001	< 0.001	< 0.001	< 0.001	< 0.001	< 0.001	< 0.001	< 0.001	< 0.001	
Control												
Dice	0.36 ± 0.19 [0.31–0.40]	0.24 ± 0.14* [0.20–0.29]	–	–	0.15 ± 0.15* [0.11–0.20]	0.16 ± 0.15* [0.11–0.20]	0.25 ± 0.19* [0.21–0.30]	0.25 ± 0.20* [0.21–0.30]	0.28 ± 0.18* [0.24–0.33]	0.28 ± 0.18* [0.23–0.32]	**0.39 ± 0.21^†^ [0.35–0.44]**	< 0.001
Hausdorff	6.67 ± 3.18 [5.75–7.60]	6.48 ± 3.75 [5.49–7.34]	–	–	8.22 ± 4.02* [7.29–9.14]	8.33 ± 4.18* [7.40–9.25]	7.37 ± 3.59* [6.45–8.30]	6.76 ± 3.60 [5.83–7.68]	7.38 ± 3.96* [6.46–8.31]	7.04 ± 4.09 [5.91–7.77]	**6.33 ± 2.91^†^ [5.41–7.26]**	< 0.001
Recall	0.52 ± 0.23 [0.46–0.58]	0.34 ± 0.19* [0.28–0.40]	–	–	0.26 ± 0.21* [0.21–0.32]	0.27 ± 0.22* [0.21–0.33]	0.37 ± 0.25* [0.31–0.43]	0.30 ± 0.24* [0.24–0.36]	0.35 ± 0.27* [0.30–0.41]	0.30 ± 0.26* [0.24–0.36]	**0.58 ± 0.26^†^ [0.52–0.64]**	< 0.001
Precision	0.37 ± 0.25* [0.32–0.43]	0.40 ± 0.25* [0.35–0.45]	–	–	0.11 ± 0.10* [0.05–0.16]	0.11 ± 0.11* [0.06–0.16]	0.28 ± 0.21* [0.23–0.34]	0.33 ± 0.24* [0.28–0.39]	0.29 ± 0.22* [0.24–0.35]	0.35 ± 0.26* [0.29–0.40]	**0.62 ± 0.25^†^ [0.56–0.67]**	< 0.001
F1 score	0.37 ± 0.19* [0.33–0.41]	0.31 ± 0.15* [0.27–0.35]	–	–	0.12 ± 0.08* [0.08–0.16]	0.13 ± 0.08* [0.09–0.17]	0.27 ± 0.15* [0.23–0.31]	0.27 ± 0.18* [0.23–0.31]	0.27 ± 0.17* [0.23–0.31]	0.28 ± 0.20* [0.24–0.32]	**0.54 ± 0.20^†^ [0.50–0.58]**	< 0.001
LOGAVD	0.30 ± 0.27 [0.20–0.41]	0.43 ± 0.40 [0.33–0.54]	–	–	0.55 ± 0.39* [0.44–0.65]	0.57 ± 0.50* [0.46–0.67]	0.42 ± 0.42 [0.32–0.53]	0.35 ± 0.35 [0.24–0.45]	0.39 ± 0.56 [0.29–0.50]	0.36 ± 0.54 [0.26–0.47]	**0.27 ± 0.30^†^ [0.16–0.37]**	< 0.001
ICC (A, 1)												
Coefficient	0.86 [0.78–0.91]	0.70 [0.18–0.87]	–	–	0.64 [0.37–0.80]	0.57 [0.30–0.74]	0.77 [0.61–0.87]	0.83 [0.73–0.89]	0.68 [0.52–0.79]	0.69 [0.53–0.80]	**0.89^†^ [0.82–0.93]**	
*p*-value	< 0.001	< 0.001	–	–	< 0.001	< 0.001	< 0.001	< 0.001	< 0.001	< 0.001	< 0.001	

Values in the table are mean ± standard deviation [95% confidence interval] from linear mixed-effects models. The lower part of the table indicates intraclass correlation coefficients and their p-values. Numbers in parentheses indicate the thresholds used in the automated algorithms. The dagger indicates the best-performing algorithm. The asterisks on the remaining algorithms denote a significant difference from the best algorithm. The best performing approach for each metric is represented in bold.

**FIGURE 1:**
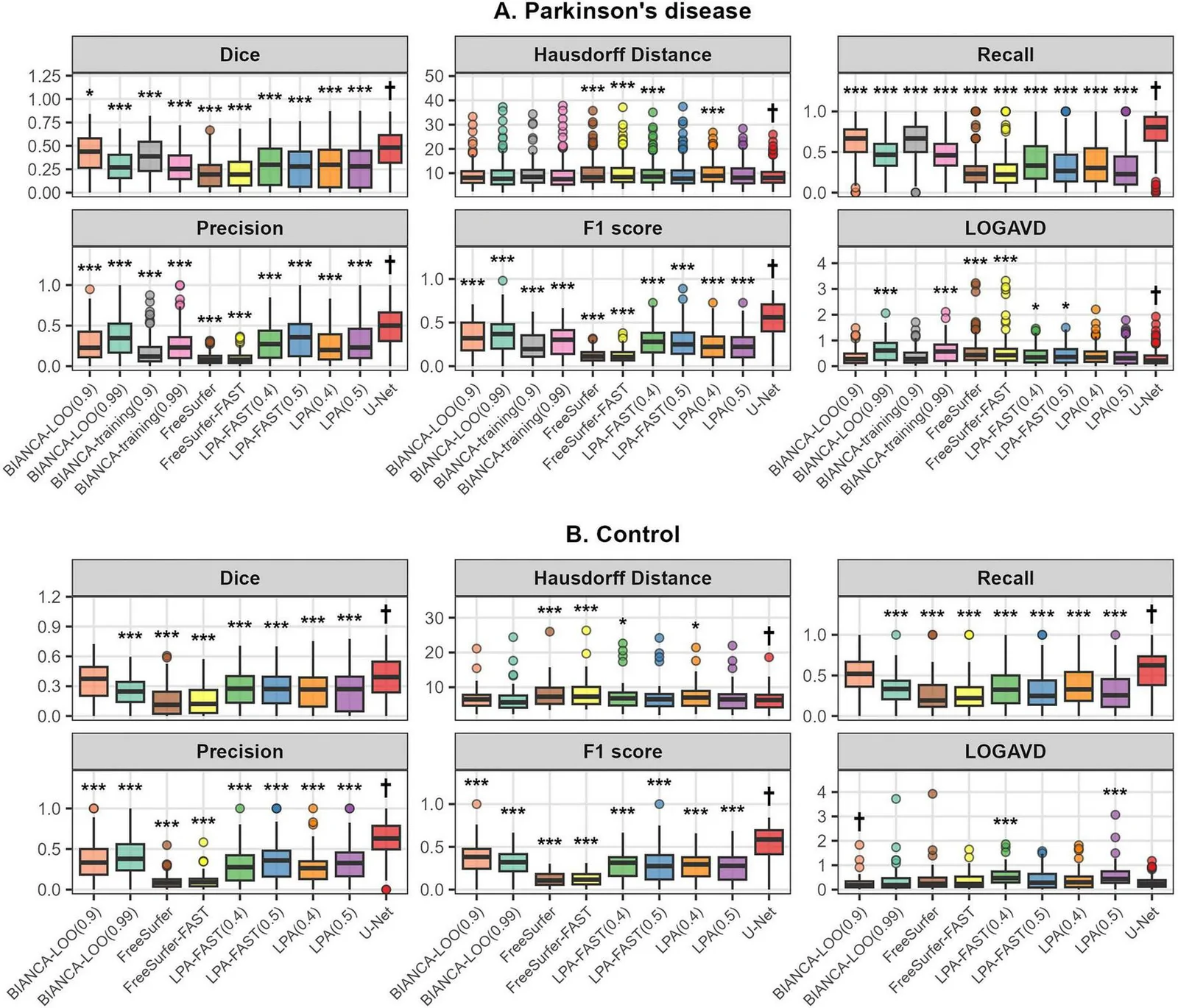
Boxplots of performance metrics across the automated approaches in the Parkinson’s disease group and control group. Boxplots show the distribution of performance metrics between automated segmentation algorithms and manual segmentation: **(A)** Parkinson’s disease group and **(B)** healthy controls. Each panel displays Dice Similarity Coefficient (higher values indicate better agreement), Hausdorff Distance (lower values indicate better agreement), recall (higher values indicate better agreement), precision (higher values indicate better agreement), F1 score (higher values indicate better agreement), and Log-transformed Absolute Volume Difference (LOGAVD; lower values indicate better agreement). The daggers indicate the best-performing algorithms within each metric and group. The asterisks indicate statistically significant differences compared to the best performer (*p < 0.05, **p < 0.01, ***p < 0.001).

**FIGURE 2:**
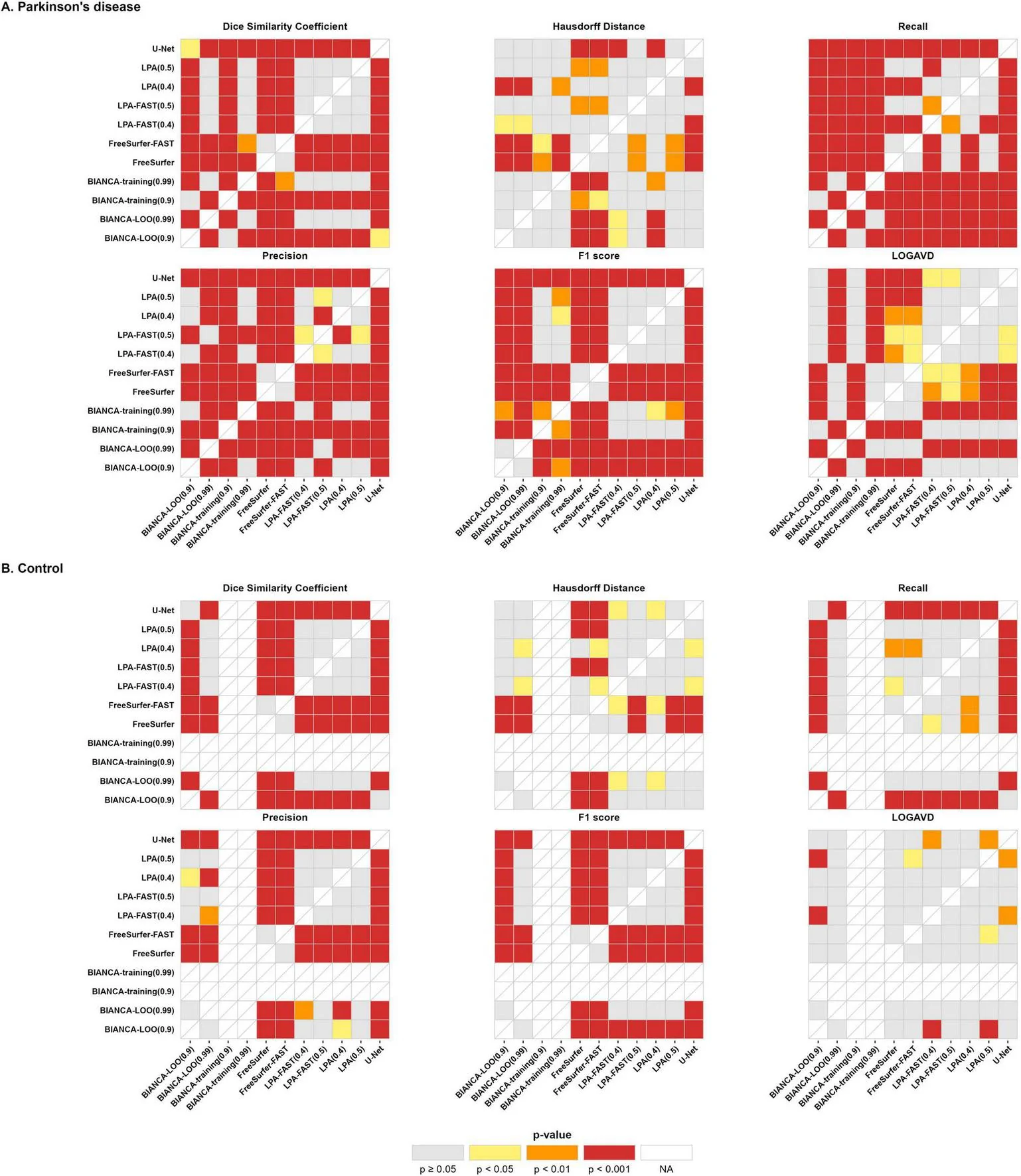
*Post-hoc* analysis of performance metrics across the automated approaches in the Parkinson’s and control group. This heatmap illustrates the pairwise *post-hoc* comparisons of automated white matter lesion segmentation algorithms in **(A)** Parkinson’s disease and **(B)** control groups. Each cell displays the statistical significance (*p*-value) of differences in segmentation performance between two algorithms based on six evaluation metrics: Dice similarity coefficient, Hausdorff distance, recall, precision, F1 score, and log-transformed absolute volume difference (LOGAVD). Color intensity encodes the level of significance, with red indicating greater statistical difference and white indicating non-significant difference.

LOGAVD was lowest (i.e., best) for U-Net in PD (0.31 ± 0.31 [0.26–0.37]) and HC (0.27 ± 0.30 [0.16–0.37]). The Hausdorff distance was also lowest (i.e., better) with U-Net in both PD and HC, with mean values of 8.89 ± 3.96 [8.19–9.59] and 6.33 ± 2.91 [5.41–7.26], respectively. However, the differences were not statistically significant from next best performing approaches.

Bland-Altman plots for comparison of the automated algorithms with manual segmentation are shown in Figure 3, visualizing the agreement between the two methods by showing how the differences vary with increasing WML volume. The WML volumes were not normally distributed, so a non-parametric approach was taken using the median, 2.5th and 97.5th percentiles.

**FIGURE 3:**
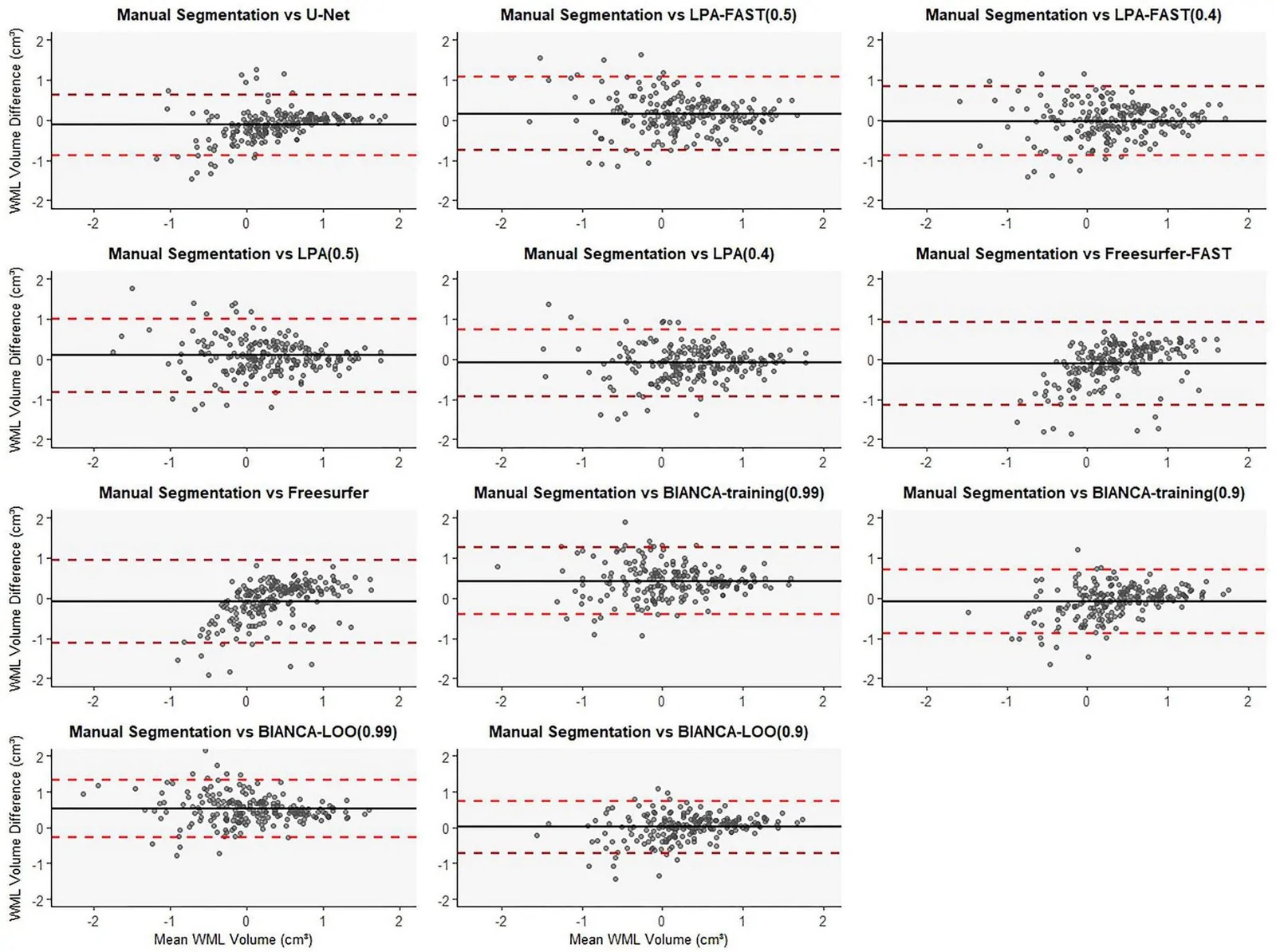
Bland-Altman plots for white matter lesion volume for the different algorithms in the Parkinson’s disease group. Each plot illustrates the agreement between an automated algorithm and the manual segmentation by showing the difference in white matter lesion (WML) volume, calculated by subtracting the automated measurement from the manually segmented volume, plotted against the mean volume of the two methods across subjects. In this non-parametric Bland–Altman analysis, the central solid line represents the median of the differences, while the dashed lines indicate the 2.5th and 97.5th percentiles.

### 3.4 Lesion load subgroup analysis

#### 3.4.1 Low WML load group

Results from the low WML load group are shown in Table 6 and Figure 4. In PD, U-Net achieved the highest Dice score (0.39 ± 0.17 [0.36–0.42]), recall (0.73 ± 0.26 [0.69–0.77]), precision (0.43 ± 0.25 [0.40–0.46]), F1 score (0.48 ± 0.21 [0.46–0.51]) and ICC (0.60 [0.37–0.74]). *Post-hoc* analyses showed all metrics were statistically significantly higher for U-Net than for all other automated approaches (Figure 5).

**TABLE 6:** Performance metrics across the automated approaches in the PD and control group with low WML loads.

Performance metric	BIANCA-LOO (0.9)	BIANCA-LOO (0.99)	BIANCA-training (0.9)	BIANCA-training (0.99)	FreeSurfer	FreeSurfer-FAST	LPA (0.4)	LPA (0.5)	LPA-FAST (0.4)	LPA-FAST (0.5)	U-Net	*p*-value
PD												
Dice	0.34 ± 0.18* [0.32–0.37]	0.22 ± 0.14* [0.19–0.25]	0.31 ± 0.17* [0.28–0.34]	0.22 ± 0.14* [0.19–0.24]	0.15 ± 0.12* [0.12–0.17]	0.16 ± 0.13* [0.13–0.18]	0.24 ± 0.19* [0.22–0.27]	0.23 ± 0.19* [0.20–0.26]	0.25 ± 0.19* [0.22–0.27]	0.23 ± 0.18* [0.20–0.26]	**0.39 ± 0.17^†^ [0.36–0.42]**	< 0.001
Hausdorff	7.28 ± 2.57* [6.82–7.74]	**6.68 ± 2.66^†^ [6.20–7.11]**	7.59 ± 2.73* [7.14–8.05]	6.72 ± 2.67 [6.26–7.18]	8.00 ± 3.11* [7.54–8.46]	7.99 ± 3.03* [7.53–8.45]	7.89 ± 2.99* [7.43–8.35]	7.32 ± 2.87* [6.87–7.78]	7.61 ± 2.81* [7.16–8.07]	7.13 ± 2.71 [6.67–7.59]	7.37 ± 2.58* [6.91–7.83]	< 0.001
Recall	0.62 ± 0.25* [0.58–0.67]	0.46 ± 0.27* [0.41–0.50]	0.64 ± 0.26* [0.60–0.69]	0.46 ± 0.25* [0.42–0.50]	0.29 ± 0.24* [0.25–0.33]	0.30 ± 0.25* [0.25–0.34]	0.37 ± 0.30* [0.32–0.41]	0.31 ± 0.28* [0.27–0.36]	0.39 ± 0.29* [0.35–0.44]	0.32 ± 0.27* [0.28–0.37]	**0.73 ± 0.26^†^ [0.69–0.77]**	< 0.001
Precision	0.23 ± 0.19* [0.20–0.26]	0.32 ± 0.24* [0.29–0.36]	0.14 ± 0.15* [0.11–0.18]	0.22 ± 0.20* [0.19–0.26]	0.07 ± 0.06* [0.04–0.11]	0.07 ± 0.06* [0.04–0.11]	0.23 ± 0.20* [0.20–0.27]	0.28 ± 0.23* [0.25–0.31]	0.25 ± 0.20* [0.22–0.29]	0.31 ± 0.25* [0.28–0.34]	**0.43 ± 0.25^†^ [0.40–0.46]**	< 0.001
F1 score	0.29 ± 0.18* [0.26–0.31]	0.31 ± 0.19* [0.29–0.34]	0.20 ± 0.15* [0.17–0.23]	0.25 ± 0.16* [0.22–0.28]	0.10 ± 0.07* [0.07–0.13]	0.10 ± 0.07* [0.08–0.13]	0.23 ± 0.16* [0.20–0.25]	0.23 ± 0.17* [0.20–0.25]	0.25 ± 0.16* [0.22–0.27]	0.24 ± 0.17* [0.22–0.27]	**0.48 ± 0.21^†^ [0.46–0.51]**	< 0.001
LOGAVD	**0.33 ± 0.29^†^ [0.27–0.40]**	0.49 ± 0.37* [0.43–0.56]	0.39 ± 0.33 [0.33–0.45]	0.46 ± 0.37* [0.40–0.52]	0.50 ± 0.51* [0.43–0.56]	0.49 ± 0.55* [0.43–0.55]	0.43 ± 0.36 [0.37–0.49]	0.37 ± 0.34 [0.31–0.44]	0.43 ± 0.36 [0.37–0.49]	0.40 ± 0.33 [0.34–0.46]	0.35 ± 0.28 [0.29–0.41]	< 0.001
ICC (A, 1)												
Coefficient	0.57 [0.45–0.67]	0.31 [-0.07–0.58]	0.46 [0.32–0.58]	0.33 [0.00–0.56]	0.18 [0.02–0.32]	0.17 [0.02–0.32]	0.50 [0.35–0.62]	0.54 [0.42–0.65]	0.47 [0.34–0.59]	0.49 [0.36–0.61]	**0.60^†^ [0.37–0.74]**	
*p-*value	< 0.001	< 0.001	< 0.001	< 0.001	0.010	0.013	< 0.001	< 0.001	< 0.001	< 0.001	< 0.001	
Control												
Dice	0.33 ± 0.17 [0.29–0.38]	0.23 ± 0.13* [0.18–0.27]	–	–	0.12 ± 0.12* [0.08–0.16]	0.13 ± 0.12* [0.09–0.17]	0.23 ± 0.17* [0.18–0.27]	0.22 ± 0.18* [0.18–0.27]	0.26 ± 0.17* [0.22–0.31]	0.26 ± 0.18* [0.22–0.30]	**0.36 ± 0.18^†^ [0.32–0.40]**	< 0.001
Hausdorff	6.11 ± 2.36 [5.39–6.83]	**5.63 ± 2.32^†^ [4.84–6.29]**	–	–	7.55 ± 3.18* [6.83–8.27]	7.68 ± 3.39* [6.96–8.40]	6.70 ± 2.69* [5.98–7.42]	6.04 ± 2.51 [5.32–6.76]	6.68 ± 2.96* [5.96–7.40]	6.19 ± 2.72 [5.28–6.73]	5.78 ± 2.25 [5.06–6.50]	< 0.001
Recall	0.53 ± 0.23 [0.47–0.60]	0.35 ± 0.19* [0.29–0.42]	–	–	0.28 ± 0.21* [0.22–0.34]	0.29 ± 0.23* [0.22–0.35]	0.39 ± 0.26* [0.32–0.45]	0.31 ± 0.25* [0.25–0.38]	0.37 ± 0.28* [0.31–0.43]	0.31 ± 0.27* [0.25–0.38]	**0.58 ± 0.26^†^ [0.51–0.64]**	< 0.001
Precision	0.33 ± 0.23* [0.28–0.39]	0.38 ± 0.25* [0.33–0.44]	–	–	0.09 ± 0.07* [0.04–0.15]	0.09 ± 0.07* [0.03–0.14]	0.26 ± 0.20* [0.21–0.32]	0.31 ± 0.24* [0.26–0.37]	0.27 ± 0.21* [0.22–0.33]	0.33 ± 0.26* [0.27–0.38]	**0.59 ± 0.25^†^ [0.54–0.65]**	< 0.001
F1 score	0.35 ± 0.19* [0.31–0.40]	0.31 ± 0.15* [0.27–0.36]	–	–	0.12 ± 0.08* [0.08–0.16]	0.12 ± 0.08* [0.08–0.16]	0.27 ± 0.16* [0.22–0.31]	0.26 ± 0.19* [0.22–0.31]	0.27 ± 0.18* [0.23–0.31]	0.28 ± 0.21* [0.24–0.32]	**0.52 ± 0.20^†^ [0.48–0.57]**	< 0.001
LOGAVD	0.26 ± 0.24 [0.15–0.37]	0.34 ± 0.29 [0.23–0.44]	–	–	0.53 ± 0.40* [0.42–0.64]	0.56 ± 0.52* [0.45–0.66]	0.41 ± 0.44 [0.30–0.52]	0.33 ± 0.35 [0.22–0.44]	0.37 ± 0.56 [0.26–0.47]	0.32 ± 0.53 [0.21–0.42]	**0.26 ± 0.23^†^ [0.15–0.36]**	< 0.001
ICC (A, 1)												
Coefficient	0.71 [0.55–0.82]	0.51 [0.04–0.76]	–	–	0.40 [-0.02 to 0.67]	0.32 [-0.01 to 0.58]	0.54 [0.27–0.72]	0.63 [0.44–0.76]	0.43 [0.19–0.62]	0.45 [0.21–0.63]	**0.78^†^ [0.64–0.86]**	
*p*-value	< 0.001	< 0.001	–	–	< 0.001	< 0.001	< 0.001	< 0.001	< 0.001	< 0.001	< 0.001	

Values in the table are mean ± standard deviation [95% confidence interval] from linear mixed-effects models. The lower part of the table indicates intraclass correlation coefficients and their p-values. Numbers in parentheses indicate the thresholds used in the automated algorithms. The dagger indicates the best-performing algorithm. The asterisks on the remaining algorithms denote a significant difference from the best algorithm. The best performing approach for each metric is represented in bold.

**FIGURE 4:**
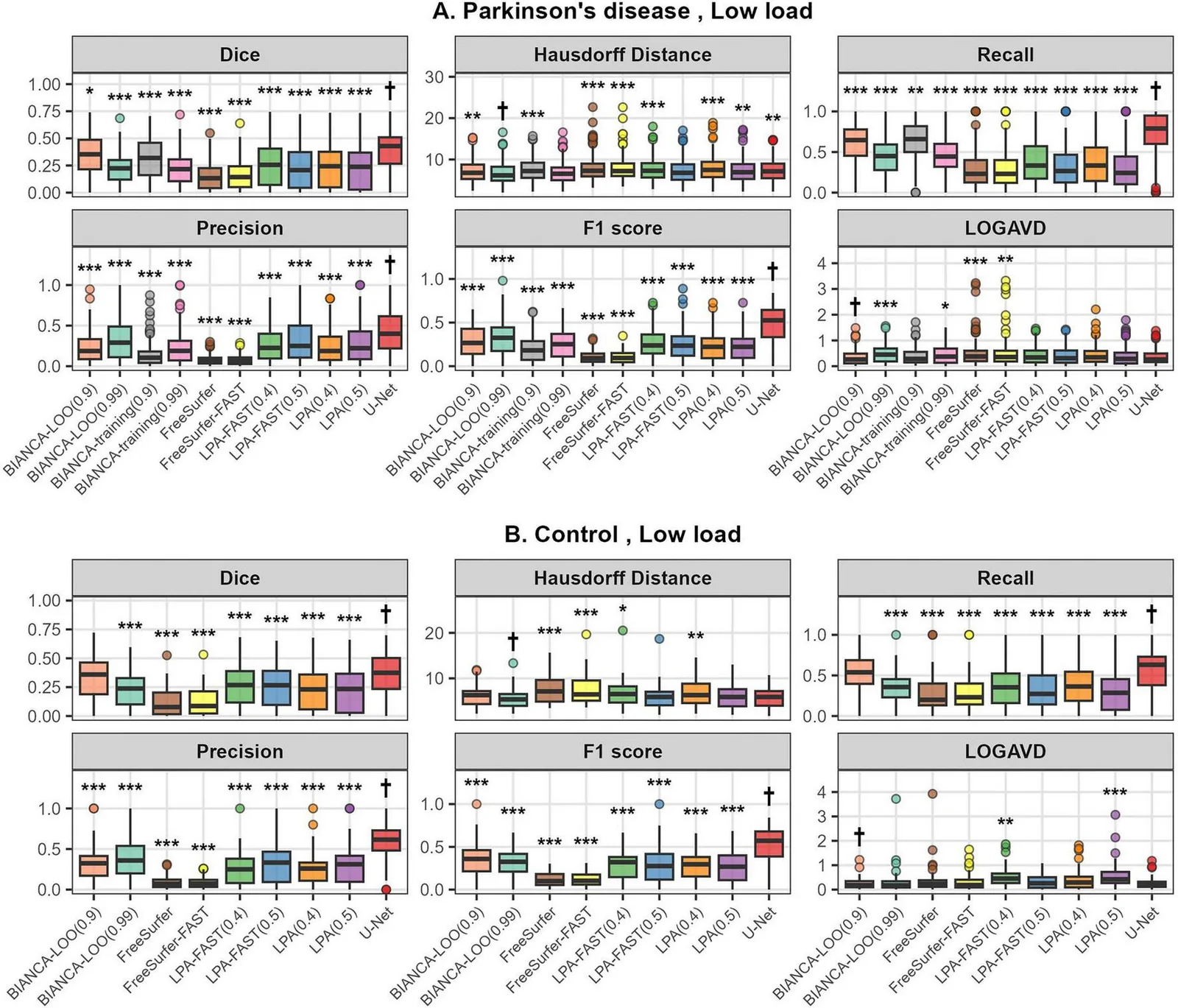
Boxplots of evaluation metrics across the automated approaches in the Parkinson’s disease group and control group for low white matter lesion load (volume < 5 cm^3^). Performance of automated segmentation algorithms were evaluated against manual segmentation using six agreement metrics: Dice Similarity Coefficient (higher values indicate better agreement), Hausdorff Distance (lower values indicate better agreement), recall (higher values indicate better agreement), precision (higher values indicate better agreement), F1 score (higher values indicate better agreement), and Log-transformed Absolute Volume Difference (LOGAVD; lower values indicate better agreement). **(A)** Parkinson’s disease patients with low lesion load, **(B)** healthy controls with low lesion load. Note that BIANCA-training algorithms were not applied to the control group. The daggers indicate the best-performing algorithms within each metric and group. The asterisks indicate statistically significant differences compared to the best-performer (*p < 0.05, **p < 0.01, ***p < 0.001).

**FIGURE 5:**
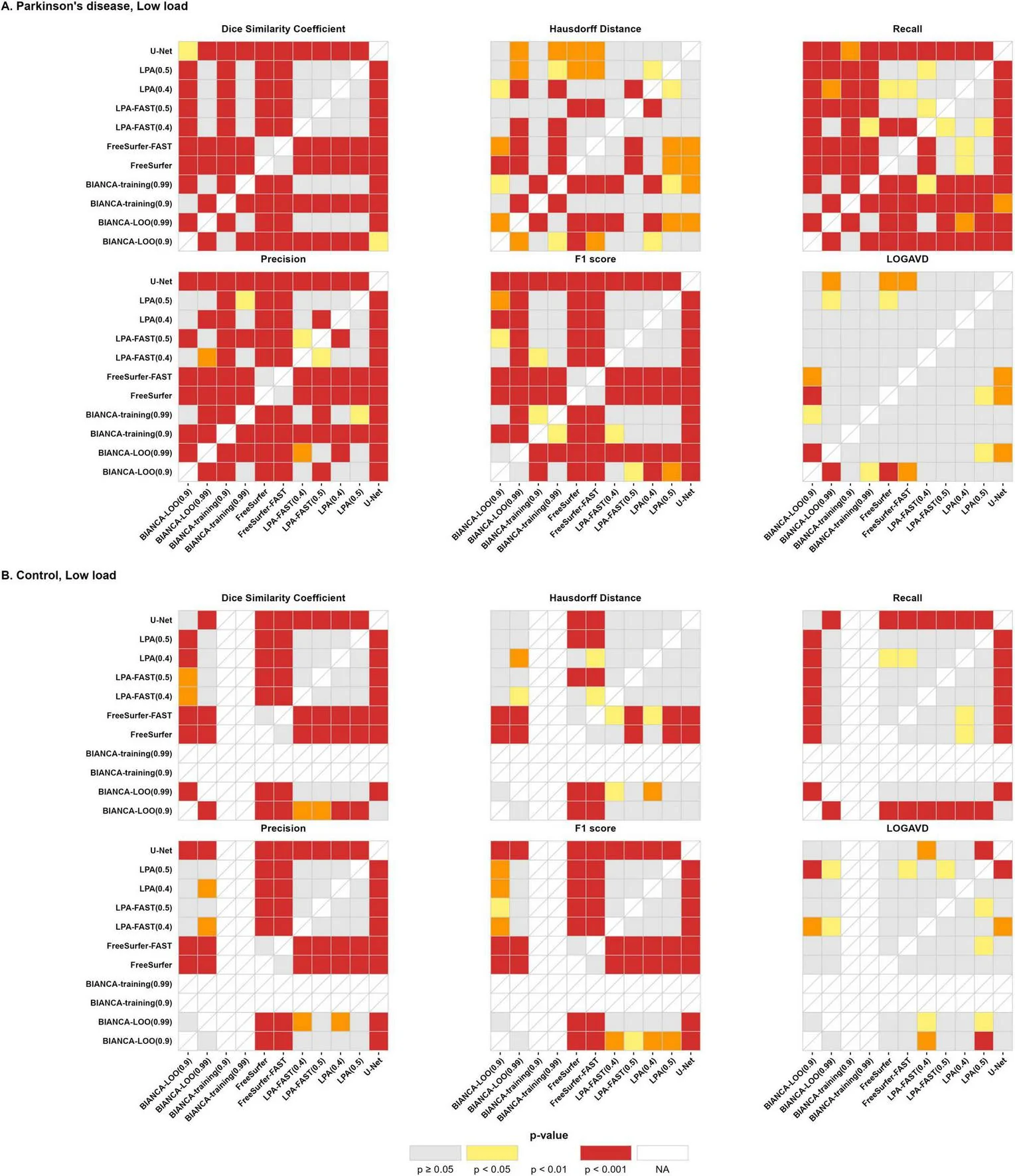
*Post-hoc* analysis of performance metrics across the automated approaches in the **(A)** Parkinson’s disease group and **(B)** control group for low white matter lesion load. This figure presents heatmaps of pairwise *post-hoc* comparisons of automated white matter lesion segmentation algorithms in subjects with low lesion load (volume < 5 cm^3^). Panel A corresponds to Parkinson’s disease patients with low lesion load, while panel B displays results from healthy controls with low lesion load. Note that BIANCA-training algorithms were not applied to the control group. Each cell displays the statistical significance (*p*-value) of differences in segmentation performance between two algorithms based on six evaluation metrics: Dice similarity coefficient, Hausdorff distance, recall, precision, F1 score, and log-transformed absolute volume difference (LOGAVD). Red indicates greater statistical difference, and white indicates non-significant difference.

In HC, U-Net also had the highest Dice score (0.36 ± 0.18 [0.32–0.40]), but the difference was not significant with BIANCA-LOO (0.9). U-Net also showed the highest recall (0.58 ± 0.26 [0.51–0.64]), precision (0.59 ± 0.25 [0.54–0.65]), F1 score (0.52 ± 0.20 [0.48–0.57]) and ICC (0.78 [0.64–0.86]). *Post-hoc* analyses showed these metrics were significantly higher for U-Net than for all other automated approaches except recall in BIANCA-LOO (0.9) (Figure 5).

BIANCA-LOO (0.9) achieved the lowest LOGAVD score in PD (0.33 ± 0.29 [0.27–0.40]) and U-Net in HC (0.26 ± 0.23 [0.15–0.36]), although these were not significantly different to those achieved by the next best performing approach (Figure 5). In both PD and HC, BIANCA-LOO (0.99) achieved the lowest Hausdorff distance (PD: 6.68 ± 2.66 [6.20–7.11], HC: 5.63 ± 2.32 [4.84–6.29]) but these were not significantly different to the next best performing approach (Figure 5).

#### 3.4.2 Medium WML load group

Results from the medium WML load group are shown in Supplementary Table 1 and Supplementary Figure 1. In PD, U-Net achieved the highest Dice score (0.61 ± 0.15 [0.56–0.67]), recall (0.76 ± 0.21 [0.70–0.83]), precision (0.61 ± 0.19 [0.55–0.66]) and F1 score (0.64 ± 0.16 [0.60–0.69]) whereas ICC was highest in BIANCA-training (0.9) (0.44 [0.12–0.67]). *Post-hoc* analyses showed all metrics for U-Net were statistically significantly higher than for all other automated approaches, bar Dice with BIANCA-LOO (0.9) and BIANCA-training (0.9) (Supplementary Figure 2). U-Net also achieved the lowest Hausdorff distance (12.31 ± 3.97 [11.00–13.63]); however, this did not significantly differ from the next best performing approach. LOGAVD was also lowest with U-Net (0.25 ± 0.42 [0.14–0.36]); this value was significantly different to BIANCA-LOO (0.99), BIANCA-training (0.99), FreeSurfer, FreeSurfer-FAST and LPA-FAST (0.5) (Supplementary Figure 2).

#### 3.4.3 High WML load group

Results from the high WML load group are shown in Supplementary Table 2 and Supplementary Figure 3. In PD, U-Net achieved the highest Dice score (0.77 ± 0.07 [0.66–0.87]), recall (0.85 ± 0.10 [0.78–0.92]), precision (0.64 ± 0.13 [0.56–0.72]), F1 score (0.72 ± 0.10 [0.66–0.78]) and ICC (0.93 [0.64–0.98]). *Post-hoc* analyses showed all metrics for U-Net were significantly higher than for all other automated approaches except for Dice with BIANCA-LOO (0.9) and BIANCA-training (0.9) and precision with BIANCA-LOO (0.99) (Supplementary Figure 4). U-Net achieved the lowest Hausdorff distance (14.00 ± 4.58 [10.30–17.70]), but the difference between the next best performing approach was not significant in *post-hoc* analysis. LOGAVD score was lowest with U-Net (0.14 ± 0.09 [0.00–0.29]), but the difference was not significant with LPA (0.4) and LPA (0.5) (Supplementary Figure 4).

### 3.5 Effect of scanner and imaging parameters

#### 3.5.1 Data source

Results of the data source sub-analysis are shown in Supplementary Table 3. In all three data sources, U-Net achieved the highest Dice, recall and F1 score and lowest LOGAVD. Precision was highest for U-Net in the C-BIG and PPMI dataset and LPA-FAST (0.5) in UPenn. ICC was highest for U-Net in C-BIG and UPenn and BIANCA-LOO (0.9) for PPMI. The lowest Hausdorff was variable, with BIANCA-LOO (0.99) in C-BIG and PPMI and LPA (0.5) in UPenn.

#### 3.5.2 Magnetic field strength

Results of the magnetic field strength sub-analysis are shown in Supplementary Table 4. For both 1.5T and 3T, U-Net achieved the highest Dice, recall, precision, F1 score, ICC and lowest LOGAVD. Hausdorff distance was lowest with BIANCA-LOO (0.99) in 1.5T and U-Net in 3T.

#### 3.5.3 FLAIR acquisition dimension

Results of the FLAIR dimension sub-analysis are shown in Supplementary Table 5. U-Net achieved the highest Dice, recall, F1 score, ICC and lowest LOGAVD for both 2D and 3D. Precision was highest with U-Net in 2D and LPA-FAST (0.5) in 3D. Hausdorff distance was lowest for BIANCA-LOO (0.99) in 2D and LPA (0.5) in 3D.

#### 3.5.4 MRI scanner manufacturer

Results of the scanner manufacturer sub-analysis are shown in Supplementary Table 6. U-Net achieved the highest Dice, recall, precision, F1 score and lowest LOGAVD for GE, Philips and Siemens. ICC was highest for LPA (0.5) in GE and U-Net in Philips and Siemens. Hausdorff distance was lowest for BIANCA-LOO (0.99) for GE, BIANCA-LOO (0.9) for Philips, and U-Net for Siemens.

### 3.6 Lobar subgroup analysis

We performed subgroup analysis according to frontal, temporal, parietal, occipital, cingulate, insular lobes, and deep white matter. The results are presented in Table 7 and Supplementary Table 7. In both groups, the Dice score was highest for U-Net in all regions except for occipital in HC in which LPA-FAST (0.5) was highest. LOGAVD was lowest with U-Net in all regions except the occipital in which BIANCA-LOO (0.9) was lowest in both PD and HC.

**TABLE 7:** Dice score and LOGAVD of automated WML measuring approaches according to lobes in the PD group.

Performance metric	BIANCA-LOO (0.9)	BIANCA-LOO (0.99)	BIANCA-training (0.9)	BIANCA-training (0.99)	FreeSurfer	FreeSurfer-FAST	LPA (0.4)	LPA (0.5)	LPA-FAST (0.4)	LPA-FAST (0.5)	U-Net	*p*-value
Frontal												
Dice	0.35 ± 0.22* [0.32–0.37]	0.22 ± 0.18* [0.18–0.24]	0.32 ± 0.22* [0.30–0.35]	0.23 ± 0.19* [0.19–0.25]	0.08 ± 0.12* [0.05–0.11]	0.08 ± 0.13* [0.05–0.10]	0.16 ± 0.21* [0.11–0.17]	0.12 ± 0.19* [0.08–0.14]	0.20 ± 0.22* [0.15–0.21]	0.15 ± 0.20* [0.10–0.16]	**0.47 ± 0.23^†^ [0.44–0.49]**	< 0.001
LOGAVD	0.21 ± 0.24* [0.16–0.25]	0.30 ± 0.33* [0.25–0.34]	0.22 ± 0.26* [0.18–0.27]	0.30 ± 0.34* [0.25–0.34]	0.37 ± 0.45* [0.32–0.41]	0.38 ± 0.45* [0.33–0.42]	0.25 ± 0.28* [0.21–0.30]	0.26 ± 0.29* [0.21–0.30]	0.25 ± 0.30* [0.21–0.30]	0.28 ± 0.33* [0.24–0.33]	**0.12 ± 0.14^†^ [0.07–0.16]**	< 0.001
Temporal												
Dice	0.17 ± 0.23* [0.12–0.18]	0.07 ± 0.15* [-0.00 to 0.07]	0.16 ± 0.22* [0.11–0.17]	0.06 ± 0.12* [-0.01 to 0.05]	0.02 ± 0.07* [-0.01 to 0.05]	0.03 ± 0.09* [-0.00 to 0.05]	0.09 ± 0.20* [0.04–0.10]	0.09 ± 0.21* [0.02–0.09]	0.13 ± 0.23* [0.07–0.13]	0.11 ± 0.21* [0.04–0.11]	**0.24 ± 0.29^†^ [0.20–0.26]**	< 0.001
LOGAVD	0.03 ± 0.09 [0.01–0.06]	0.04 ± 0.12 [0.02–0.07]	0.04 ± 0.09 [0.01–0.06]	0.05 ± 0.12 [0.02–0.07]	0.14 ± 0.37* [0.12–0.17]	0.15 ± 0.39* [0.12–0.17]	0.05 ± 0.11 [0.02–0.07]	0.04 ± 0.11 [0.01–0.07]	0.04 ± 0.11 [0.02–0.07]	0.04 ± 0.11 [0.01–0.07]	**0.03 ± 0.07^†^ [0.00–0.06]**	< 0.001
Parietal												
Dice	0.33 ± 0.25* [0.29–0.36]	0.23 ± 0.21* [0.19–0.25]	0.32 ± 0.25* [0.29–0.35]	0.23 ± 0.20* [0.19–0.25]	0.11 ± 0.16* [0.08–0.14]	0.11 ± 0.16* [0.08–0.14]	0.17 ± 0.25* [0.12–0.19]	0.15 ± 0.24* [0.10–0.16]	0.20 ± 0.24* [0.15–0.21]	0.16 ± 0.21* [0.11–0.17]	**0.40 ± 0.25^†^ [0.36–0.42]**	< 0.001
LOGAVD	0.13 ± 0.16 [0.09–0.17]	0.19 ± 0.26* [0.16–0.23]	0.14 ± 0.17 [0.10–0.18]	0.20 ± 0.26* [0.16–0.23]	0.26 ± 0.38* [0.23–0.30]	0.27 ± 0.39* [0.23–0.30]	0.19 ± 0.26* [0.15–0.23]	0.20 ± 0.27* [0.16–0.23]	0.19 ± 0.26* [0.15–0.23]	0.20 ± 0.28* [0.17–0.24]	**0.10 ± 0.12^†^ [0.06–0.13]**	< 0.001
Occipital												
Dice	0.14 ± 0.20* [0.10–0.15]	0.06 ± 0.12* [-0.00 to 0.05]	0.13 ± 0.19* [0.09–0.15]	0.06 ± 0.11* [-0.01 to 0.04]	0.05 ± 0.10* [0.02–0.08]	0.05 ± 0.10* [0.02–0.07]	0.17 ± 0.25 [0.13–0.19]	0.17 ± 0.24* [0.12–0.18]	0.19 ± 0.25 [0.15–0.21]	0.18 ± 0.24 [0.13–0.19]	**0.20 ± 0.25^†^ [0.17–0.23]**	< 0.001
LOGAVD	**0.10 ± 0.15^†^ [0.07–0.13]**	0.11 ± 0.19 [0.08–0.14]	0.10 ± 0.14 [0.07–0.13]	0.11 ± 0.19 [0.08–0.14]	0.21 ± 0.32* [0.18–0.24]	0.21 ± 0.36* [0.18–0.24]	0.15 ± 0.22 [0.12–0.18]	0.13 ± 0.20 [0.10–0.16]	0.17 ± 0.21* [0.13–0.20]	0.14 ± 0.19 [0.11–0.17]	0.19 ± 0.18* [0.16–0.22]	< 0.001
Cingulate												
Dice	0.21 ± 0.24* [0.16–0.22]	0.11 ± 0.16* [0.04–0.10]	0.23 ± 0.24* [0.18–0.24]	0.12 ± 0.17* [0.05–0.11]	0.05 ± 0.12* [0.02–0.08]	0.05 ± 0.12* [0.02–0.08]	0.14 ± 0.23* [0.10–0.16]	0.12 ± 0.22* [0.07–0.13]	0.13 ± 0.19* [0.09–0.14]	0.11 ± 0.18* [0.06–0.12]	**0.32 ± 0.29^†^ [0.26–0.32]**	< 0.001
LOGAVD	0.04 ± 0.09 [0.03–0.06]	0.06 ± 0.13* [0.04–0.07]	0.04 ± 0.09 [0.02–0.06]	0.05 ± 0.13* [0.04–0.07]	0.08 ± 0.14* [0.07–0.10]	0.09 ± 0.16* [0.08–0.11]	0.07 ± 0.12* [0.06–0.09]	0.06 ± 0.10* [0.05–0.08]	0.05 ± 0.09* [0.04–0.07]	0.05 ± 0.10* [0.03–0.07]	**0.02 ± 0.05^†^ [0.01–0.04]**	< 0.001
Insular												
Dice	0.31 ± 0.27* [0.26–0.33]	0.22 ± 0.24* [0.14–0.22]	0.29 ± 0.27* [0.24–0.31]	0.23 ± 0.24* [0.17–0.24]	0.07 ± 0.15* [0.01–0.08]	0.07 ± 0.14* [0.01–0.08]	0.16 ± 0.24* [0.09–0.16]	0.13 ± 0.21* [0.05–0.13]	0.18 ± 0.24* [0.10–0.18]	0.15 ± 0.22* [0.06–0.14]	**0.42 ± 0.29^†^ [0.35–0.42]**	< 0.001
LOGAVD	0.04 ± 0.08[0.03–0.06]	0.06 ± 0.12* [0.04–0.07]	0.05 ± 0.08[0.03–0.06]	0.06 ± 0.11* [0.04–0.07]	0.10 ± 0.21* [0.08–0.12]	0.09 ± 0.21* [0.08–0.11]	0.06 ± 0.09* [0.04–0.07]	0.06 ± 0.10* [0.04–0.07]	0.06 ± 0.13* [0.04–0.08]	0.06 ± 0.14* [0.04–0.08]	**0.03 ± 0.05^†^ [0.01–0.05]**	< 0.001
DWM												
Dice	0.46 ± 0.23* [0.43–0.49]	0.30 ± 0.18* [0.27–0.33]	0.45 ± 0.23* [0.41–0.48]	0.31 ± 0.19* [0.27–0.34]	0.29 ± 0.21* [0.26–0.32]	0.30 ± 0.22* [0.27–0.33]	0.35 ± 0.27* [0.32–0.38]	0.33 ± 0.27* [0.30–0.37]	0.40 ± 0.25* [0.37–0.43]	0.38 ± 0.25* [0.35–0.41]	**0.52 ± 0.23^†^ [0.48–0.55]**	< 0.001
LOGAVD	0.26 ± 0.23 [0.22–0.30]	0.44 ± 0.33* [0.40–0.48]	0.28 ± 0.24 [0.24–0.31]	0.43 ± 0.35* [0.39–0.47]	0.34 ± 0.27* [0.31–0.38]	0.37 ± 0.31* [0.33–0.41]	0.29 ± 0.28* [0.25–0.33]	0.31 ± 0.27* [0.27–0.35]	0.32 ± 0.26* [0.28–0.36]	0.33 ± 0.27* [0.29–0.37]	**0.21 ± 0.24^†^ [0.17–0.25]**	< 0.001

Values are expressed as mean ± standard deviation (95% confidence interval). Numbers in parentheses indicate the thresholds used in the automated algorithms. The dagger indicates the best-performing algorithm. The asterisks on the remaining algorithms denote a significant difference from the best algorithm. The best performing approach for each metric is represented in bold.

### 3.7 Correlation with age and visual rating scales

#### 3.7.1 Correlation of outputs using age

In the PD group, WML volumes from all the automated segmentation approaches showed correlations with age (Supplementary Table 8). LPA (0.5) had the highest Pearson correlation coefficient (*r* = 0.489, *p* < 0.001), which was higher than that of manual segmentation (*r* = 0.434, *p* < 0.001).

#### 3.7.2 Correlation of outputs using visual rating scales

In the PD group, the WML volumes from the manual segmentation and from all the automated segmentation approaches were well correlated to both Fazekas and Wahlund score, after adjusting for age (Supplementary Table 9). U-Net had the highest partial correlation coefficient among the automated approaches (Fazekas *r* = 0.805, *p* < 0.001, Wahlund *r* = 0.811, *p* < 0.001) with similar values to that of the manual segmentation (Fazekas *r* = 0.863, *p* < 0.001, Wahlund *r* = 0.874, *p* < 0.001).

### 3.8 Visual assessment of WML segmentations

All outputs from the automated approaches were visually compared to the manual segmentations. All approaches produced some minor inaccuracies; however, U-Net and BIANCA tended to produce the most accurate maps overall. Small deep white matter lesions were often missed with the BIANCA approach after thresholding whilst U-Net was noted to detect both periventricular and deep white matter lesions well but had a tendency to misidentify the optic radiation as WML (Figure 6).

**FIGURE 6:**
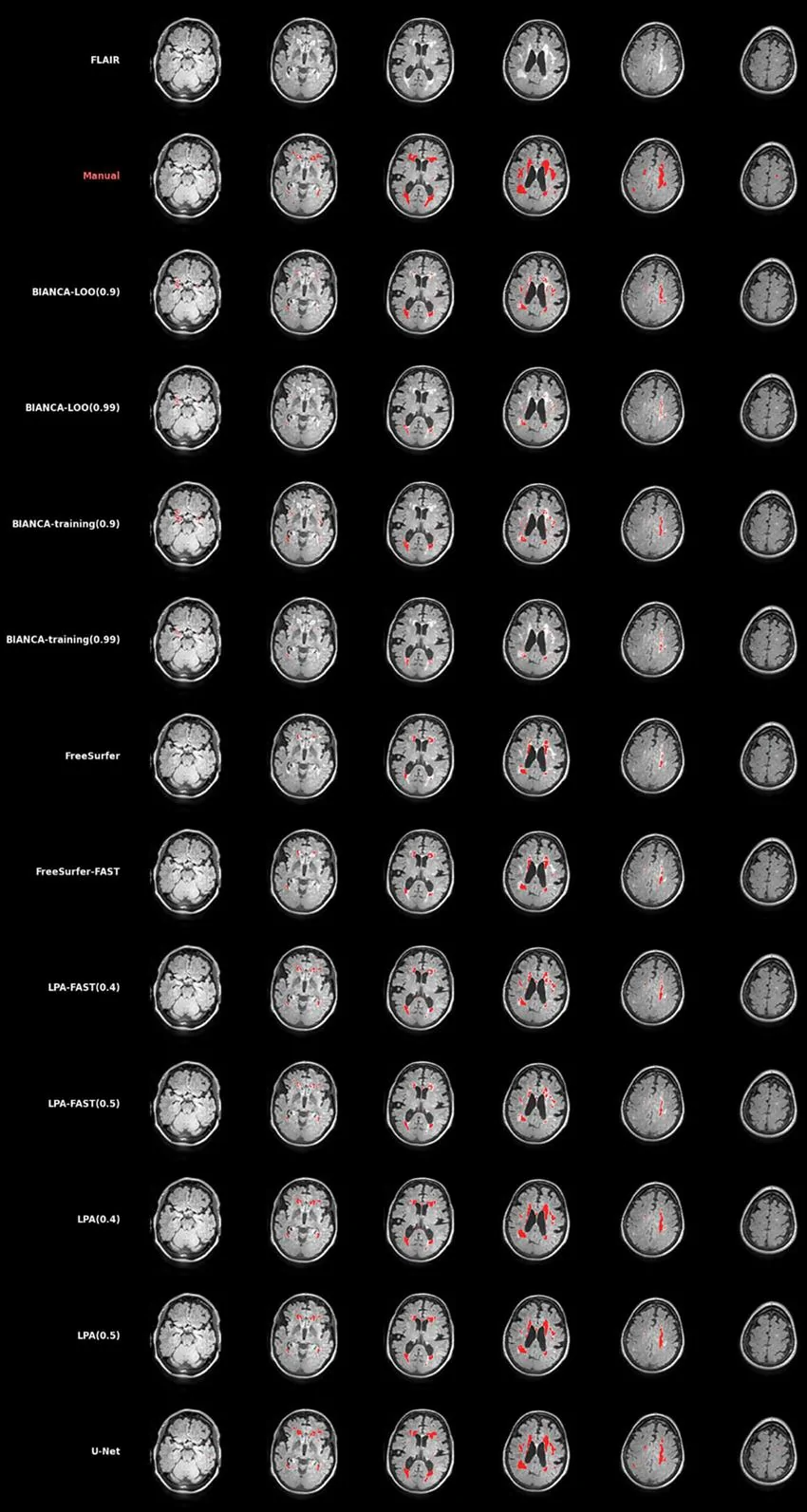
An example segmentation from each of the automated approaches. Top row shows FLAIR MRI slices from a single example subject. The segmentation provided by each automated approach is shown in subsequent rows.

All approaches also produced significant errors in a proportion of the segmentation outputs. A summary of the common significant errors and their frequency are shown in Table 8. These scans were not excluded from the analysis of performance metrics to allow the metrics to reflect the accuracy of each approach, but in practice would be excluded during a quality check step due to complete failure of the automated approach. We failed to identify any particular pattern in the original images or scanner and imaging parameters which led to these errors, emphasizing the need for quality checking of all outputs, as no automated approach is foolproof.

**TABLE 8:** Summary of significant common errors and frequency per automated approach identified from visual assessment.

	BIANCA	FreeSurfer	LPA	U-Net
Overestimation of WML	0	13	15	4
Missed WML	14	2	3	7
Segmentation of non-white matter	0	7	3	7
Mismatched segmentation and original image	3	1	3	0
Total errors	17	23	24	18

Values expressed as number of significant errors seen from each automated approach. The errors seen for BIANCA, FreeSurfer and LPA were present regardless of training model and threshold.

## 4 Discussion

The purpose of this study was to compare and identify an optimal WML automated segmentation approach to use on multi-site data in PD, which is robust to data from various scanners with heterogeneous acquisition parameters. Of the automated approaches assessed, U-Net was highest achieving overall, relative to the gold standard of manual segmentation, reflected by the majority of comparisons showing most favorable Dice and ICC values for spatial and volumetric agreement and consistently more favorable F1, recall and precision scores, reflecting a good balance of true positives and false negatives. This general pattern was seen in both PD and HC, as well as with various scanner and imaging parameters as demonstrated in sub-analyses.

U-Net consistently outperformed other approaches in the full PD and HC analysis; however, when sub-grouped into lesion loads, there were some occasions where other approaches showed superior performance. In the low WML load group, Hausdorff distance was lowest in BIANCA-LOO (0.99) and LOGAVD in BIANCA-LOO (0.9) for PD. As Hausdorff distance is highly sensitive to outliers (Taha and Hanbury, 2015), even a single outlier can substantially impact the calculated distance. As many of our scans had dot-like small lesions, this might have led to a misleading comparison between approaches in the *post-hoc* analysis. Additionally, overestimation of the optic radiation with U-Net, which was identified in visual assessment, may account for the poorer performance on LOGAVD. Even a small absolute difference in volume can substantially impact LOGAVD score, as this logarithmic metric compresses large differences and expands small ones, potentially exaggerating the perceived error. Hence it is important to combine various performance metrics to not only understand volumetric and spatial agreement, but what drives them.

Although BIANCA-LOO performed best on certain metrics discussed above, the BIANCA-LOO approach is not scalable in multi-site studies as it requires manual labeling of all subjects to create subject-specific training sets. Instead, the BIANCA-LOO analysis can be interpreted as an approximate upper bound on the performance achievable by BIANCA when all available manually labeled data are used for training. In practice, BIANCA’s performance depends strongly on the quality and representativeness of the training set. Future work should investigate the impact of larger and more heterogeneous training datasets.

In the lobar subgroup analysis, U-Net also had the highest Dice and lowest LOGAVD in both groups across all lobes, except the occipital lobe, reflecting good spatial and volumetric agreement with manual segmentation. The tendency of U-Net to misclassify the optic radiation as WML may explain its relatively weaker performance in the occipital lobe. Studies assessing WML in PD have produced conflicting results which may be attributable to differences in segmentation approach, for example under-identifying lesions in clinically eloquent brain regions (Butt et al., 2021). Therefore, an approach that accurately detects the location of WML, regardless of which lobe lesions are located in, may reduce the risk of missing clinically relevant lesions in PD.

Of the approaches we tested, only U-Net and BIANCA have been specifically developed for segmentation of WML of vascular origin. FreeSurfer performs general tissue type classification, and LPA was trained on 53 subjects with multiple sclerosis, although it has been successfully applied to WML of vascular origin in other conditions (Schmidt et al., 2012). Our results showed U-Net and BIANCA to generally outperform the other methods, suggesting this specificity for vascular lesions may result in better performance. In particular, the superior performance of U-Net is likely due to its architecture (Park et al., 2021). As an ensemble method with highlighting foregrounds, it has greater ability to detect the boundaries of lesions and reduce partial volume effects, which are a particular issue with small lesions.

Our findings in PD are largely consistent with current literature on the topic in other populations. The MICCAI WMH Segmentation Challenge aimed to compare the segmentation of WML of presumed vascular origin on MR images using automated approaches in patients from five memory and aging cohorts. Results revealed no single method excelled in all metrics, yet U-Net generally performed better at various lesion loads (Kuijf et al., 2019). Results also showed that ensemble methods (such as U-Net, which we implemented) outperformed other convolutional neural networks (CNN), highlighting that smaller WML were often missed by the other CNN methods and methods that were less “location sensitive” were better in this regard (Ghafoorian et al., 2016; Kuijf et al., 2019). As both the size and shape of WML are clinically impactful, it is essential to select an approach that does not miss these smaller lesions (de Bresser et al., 2018). Torres-Simon et al. demonstrated in healthy aging participants that although LPA and BIANCA outperformed other approaches tested (they did not include U-Net in their comparison), they failed to consistently segment lesions smaller than 8 mm^3^. They argued deep learning approaches show promise for small lesion segmentation due to the utility of multiscale deep features (Park et al., 2021; Torres-Simon et al., 2024). Studies comparing BIANCA and other k-nearest neighbor techniques in healthy aging and cognitively impaired populations concluded BIANCA/k-NN-TTP followed by LST-LPA outperformed other methods tested, including in longitudinal data, while FreeSurfer consistently underestimated WML volumes (Heinen et al., 2019; Ribaldi et al., 2021; Hotz et al., 2022). Interestingly, a recent review of WML automated approaches found no evidence that deep learning was superior to other supervised or unsupervised techniques; however, this discrepancy likely arises as the inclusion criteria encompassed both WML of vascular origin and inflammatory WML such as seen in multiple sclerosis. Most of the studies included in the review did not use multi-site data (Balakrishnan et al., 2021).

In this study, we included data from various scanners and acquisition parameters to reflect consortium data which is often variable. U-Net performed well despite these differences. In addition, for an automated approach to be amenable to consortium data there are several logistical considerations. Various sites will have differing computational facilities and skillsets. Ideally, BIANCA requires a training set that is representative of the population to which it is being applied, hence a training set may require combining labeled samples from different sites, increasing the level of manual input required compared to U-Net. Nonetheless, U-Net is computationally demanding and has a longer running time than BIANCA. Additionally, all automated approaches require the installation of software packages, file preparation and pre-processing steps, as well as manual quality control. Future work should consider a containerized approach to large multi-site analysis i.e., a pipeline that includes all the code, tools and dependencies required to output the WML segmentations in a standardized way.

As expected, all automated approaches showed some correlation between WML and age and visual rating scales. However, simple correlation does not necessarily reflect spatial or neuroanatomical agreement of the approaches, as reflected by our performance metrics. The extent to which WML segmentations reflect meaningful pathological or clinical correlates remains uncertain and was beyond the scope of this study primarily because these associations are still unclear. However, both burden and precise location of WML are now thought to be important (Lampe et al., 2017; Coenen et al., 2022), therefore the assumption made by the authors is that a technique that produces volumetric and spatially accurate WML segmentations in heterogeneous data would allow for clinical correlations to be elucidated in further large-scale analyses. Work is in progress on implementing this pipeline to other disease states with vascular pathology, such as stroke, with initial promising results.

There are also other limitations to our study. Its cross-sectional design precluded assessment of longitudinal consistency or progression sensitivity, which are important for evaluating the practical applicability of these methods in clinical and research settings. Some longitudinal studies have shown “outlier intervals,” where factors such as atrophy and co-morbidities affected outcomes of approaches such as BIANCA (Hotz et al., 2022). However, recent studies suggest deep learning techniques such as U-Net may actually help in modeling longitudinal multi-modal MRI changes, including WML (Rachmadi et al., 2020; Gong et al., 2024). We also acknowledge that other automated segmentation approaches have been developed since this study was conducted. Tsuchida et al. (2023) found that their SHIVA-WMH tool produced more accurate segmentations than U-Net-pgs; however, this comparison examined only 21 healthy subjects in two cohorts, with all scans collected on a 3T scanner with 1 mm isotropic voxels (for T1w images) and 1.00 × 1.00 × 1.00mm^3^ to 1.05 × 1.00 × 1.00mm^3^ voxels (for FLAIR images). Additionally, U-Net-pgs was used without retraining the model on the same training data used by SHIVA-WMH (Tsuchida et al., 2023).

Our study also has several strengths. To our knowledge, it is the first attempt to evaluate automated WML algorithms specifically in PD, comparing their outputs to manual segmentations with whole brain and lobar assessments. Associations with age and visual rating scales were also made, allowing for comparison to alternative approaches. We used heterogeneous data with varying acquisition parameters and WML load and location, reflecting the real-world clinical environment and consortium data. We demonstrated promise for an approach that produces accurate WML segmentations despite these differing parameters across all brain regions, indicating U-Net’s potential utility for large, multi-site data analyses in PD and controls, with potential for detecting WML of vascular origin in other conditions.

## 5 Conclusion

In summary, accurate and representative WML segmentations are key to analysis of the clinical impact of WML of presumed vascular origin. Optimization of an analysis approach on multi-site WML data specifically in a PD population was lacking. This paper outlines our attempts to select an optimal WML segmentation approach, in multi-site data, by comparing WML automated segmentation methods against the gold standard of manual segmentations. Overall, we found U-Net as the most suitable automated method for WML segmentation, of those tested, in PD and controls, consistently outperforming other algorithms across various lesion loads and in most lobar regions. Although BIANCA-LOO also performed to a high standard, this requires manual segmentation of all subjects in the dataset, which is not feasible for large-scale analyses. No automated approach was without limitations, therefore manual quality control of the outputs from all the automated approaches is essential. Future work should focus on validating these findings in independent cohorts and other disease states, ultimately allowing for incorporation of U-Net into a containerized imaging pipeline to streamline large-data analyses of WML patterns.

## Data Availability

The raw data supporting the conclusions of this article will be made available by the authors, without undue reservation.

## Footnotes

1
https://www.ppmi-info.org/

2
https://www.med.upenn.edu/pdmdc/

3
www.statistical-modelling.de/lst.html
