# Supplementary material for: Comparison of automated white matter lesion segmentation approaches for use in large, multi-site data analyses in Parkinson’s disease
Source: Front Neurosci. 2026 Sep 24;20:1904361. doi: 10.3389/fnins.2026.1904361 (PMC13647306; doi:10.3389/fnins.2026.1904361)
Supplement: Supplementary file 1 [file Data_Sheet_1.docx]

**Supplementary Table 1: Performance metrics across the automated approaches in the Parkinson’s disease group with medium white matter lesion load.**

Values in the table are mean ± standard deviation [95% confidence interval] from linear mixed-effects models. The lower part of the table indicates intraclass correlation coefficients and their p-values. Numbers in parentheses indicate the thresholds used in the automated algorithms. The dagger indicates the best-performing algorithm. The asterisks on the remaining algorithms denote a significant difference from the best algorithm. The best performing approach for each metric is represented in bold.

|  | BIANCA  -LOO  (0.9) | BIANCA  -LOO  (0.99) | BIANCA -training (0.9) | BIANCA -training  (0.99) | FreeSurfer | FreeSurfer -FAST | LPA  (0.4) | LPA  (0.5) | LPA  -FAST  (0.4) | LPA  -FAST  (0.5) | U-Net | p-value |
| --- | --- | --- | --- | --- | --- | --- | --- | --- | --- | --- | --- | --- |
| Dice | 0.58 ± 0.11  [0.52–0.63] | 0.40 ± 0.13* [0.34–0.45] | 0.55 ± 0.12 [0.50–0.61] | 0.39 ± 0.13* [0.34–0.45] | 0.34 ± 0.14* [0.28–0.39] | 0.34 ± 0.15* [0.29–0.40] | 0.41 ± 0.24* [0.35–0.46] | 0.40 ± 0.25* [0.34–0.45] | 0.44 ± 0.21* [0.38–0.49] | 0.41 ± 0.21* [0.36–0.47] | **0.61 ± 0.15† [0.56–0.67]** | < 0.001 |
| Hausdorff | 12.75 ± 4.00  [11.43–14.06] | 13.47 ± 4.23* [12.15–14.78] | 13.00 ± 4.12  [11.68–14.32] | 13.64 ± 4.46* [12.33–14.96] | 13.75 ± 4.56*  [12.43–15.07] | 13.71 ± 4.70* [12.40–15.03] | 13.67 ± 3.45* [12.35–14.99] | 13.32 ± 3.52 [12.01–14.64] | 13.10 ± 3.88 [11.78–14.41] | 12.99 ± 3.97 [11.68–14.31] | **12.31 ± 3.97†  [11.00–13.63]** | < 0.001 |
| Recall | 0.69 ± 0.19 [0.63–0.76] | 0.53 ± 0.19* [0.46–0.60] | 0.69 ± 0.20 [0.62–0.75] | 0.52 ± 0.20* [0.45–0.59] | 0.22 ± 0.10* [0.16–0.29] | 0.22 ± 0.11* [0.16–0.29] | 0.37 ± 0.29* [0.31–0.44] | 0.33 ± 0.28* [0.26–0.39] | 0.44 ± 0.24* [0.37–0.51] | 0.38 ± 0.23* [0.31–0.45] | **0.76 ± 0.21†**  **[0.70–0.83]** | < 0.001 |
| Precision | 0.34 ± 0.20* [0.28–0.40] | 0.45 ± 0.23* [0.39–0.50] | 0.21 ± 0.15* [0.16–0.27] | 0.32 ± 0.20* [0.26–0.38] | 0.14 ± 0.08* [0.08–0.19] | 0.13 ± 0.09* [0.07–0.19] | 0.30 ± 0.22* [0.25–0.36] | 0.33 ± 0.22* [0.27–0.38] | 0.40 ± 0.19* [0.34–0.46] | 0.44 ± 0.19* [0.38–0.50]) | **0.61 ± 0.19†**  **[0.55–0.66]** | < 0.001 |
| F1 score | 0.41 ± 0.19* [0.37–0.46] | 0.43 ± 0.16* [0.39–0.48] | 0.29 ± 0.16* [0.25–0.34] | 0.36 ± 0.16 * [0.31–0.40] | 0.15 ± 0.07* [0.10–0.20] | 0.14 ± 0.08* [0.10–0.19] | 0.28 ± 0.18* [0.23–0.32] | 0.26 ± 0.17* [0.22–0.31] | 0.36 ± 0.13* [0.31–0.40] | 0.34 ± 0.13* [0.30–0.39] | **0.64 ± 0.16†**  **[0.60–0.69]** | < 0.001 |
| LOGAVD | 0.34 ± 0.28  [0.23–0.44] | 0.92 ± 0.37* [0.81–1.02] | 0.29 ± 0.26  [0.18–0.39] | 0.86 ± 0.39* [0.75–0.97] | 0.59 ± 0.34* [0.49–0.70] | 0.59 ± 0.34* [0.48–0.69] | 0.42 ± 0.28  [0.31–0.53] | 0.43 ± 0.32  [0.32–0.54] | 0.42 ± 0.28  [0.31–0.52] | 0.48 ± 0.36* [0.38–0.59] | **0.25 ± 0.42†**  **[0.14–0.36]** | < 0.001 |
| ICC (A, 1) |  |  |  |  |  |  |  |  |  |  |  |  |
| Coefficient | 0.40  [0.00–0.67] | 0.10  [-0.06–0.35] | **0.44† [0.12–0.67]** | 0.11  [-0.07–0.37] | 0.17  [-0.08–0.42] | 0.21  [-0.06–0.46] | 0.39  [0.08–0.62] | 0.37  [0.08–0.61] | 0.41  [0.13–0.64] | 0.34  [-0.00–0.60] | 0.32  [0.03–0.57] |  |
| p-value | < 0.001 | 0.002 | < 0.001 | 0.003 | 0.062 | 0.041 | 0.008 | 0.005 | 0.003 | 0.002 | 0.012 |  |

**Supplementary Table 2: Performance metrics across the automated approaches in the Parkinson’s disease group with high white matter lesion load.**

Values in the table are mean ± standard deviation [95% confidence interval] from linear mixed-effects models. The lower part of the table indicates intraclass correlation coefficients and their p-values. Numbers in parentheses indicate the thresholds used in the automated algorithms. The dagger indicates the best-performing algorithm. The asterisks on the remaining algorithms denote a significant difference from the best algorithm. The best performing approach for each metric is represented in bold.

|  | BIANCA  -LOO  (0.9) | BIANCA  -LOO  (0.99) | BIANCA  -training  (0.9) | BIANCA  -training  (0.99) | FreeSurfer | FreeSurfer  -FAST | LPA  (0.4) | LPA  (0.5) | LPA  -FAST  (0.4) | LPA  -FAST  (0.5) | U-Net | p-value |
| --- | --- | --- | --- | --- | --- | --- | --- | --- | --- | --- | --- | --- |
| Dice | 0.68 ± 0.10  [0.58–0.79] | 0.48 ± 0.12* [0.38–0.58] | 0.66 ± 0.12  [0.56–0.77] | 0.47 ± 0.13* [0.37–0.57] | 0.44 ± 0.11*  [0.34–0.54] | 0.46 ± 0.10* [0.36–0.56] | 0.44 ± 0.36* [0.33–0.54] | 0.43 ± 0.35* [0.32–0.53] | 0.37 ± 0.30*  [0.27–0.47] | 0.34 ± 0.28* [0.23–0.44] | **0.77 ± 0.07† [0.66–0.87]** | <0.001 |
| Hausdorff | 16.11 ± 7.63  [12.41–19.81] | 19.27 ± 8.94* [15.57–22.97] | 16.69 ± 8.17  [12.99–20.39] | 19.38 ± 9.43* [15.69–23.08] | 19.07 ± 7.57* [15.37–22.77] | 18.68 ± 7.56* [14.98–22.38] | 17.50 ± 4.94* [13.81–21.20] | 17.65 ± 5.36* [13.95–21.35] | 19.31 ± 7.10* [15.61–23.00] | 19.97 ± 7.76* [16.27–23.67] | **14.00 ± 4.58† [10.30–17.70]** | <0.001 |
| Recall | 0.64 ± 0.16* [0.57–0.71] | 0.45 ± 0.13* [0.38–0.52] | 0.64 ± 0.13* [0.57–0.71] | 0.44 ± 0.14* [0.37–0.51] | 0.20 ± 0.10* [0.13–0.27] | 0.19 ± 0.10* [0.12–0.26] | 0.27 ± 0.18* [0.20–0.34] | 0.23 ± 0.17* [0.16–0.30] | 0.25 ± 0.19* [0.18–0.32] | 0.22 ± 0.17* [0.15–0.29] | **0.85 ± 0.10† [0.78–0.92]** | <0.001 |
| Precision | 0.44 ± 0.15* [0.36–0.52] | 0.51 ± 0.14  [0.43–0.59] | 0.30 ± 0.14* [0.22–0.38] | 0.38 ± 0.12* [0.29–0.46] | 0.16 ± 0.07* [0.08–0.25] | 0.16 ± 0.08* [0.08–0.24] | 0.28 ± 0.21* [0.20–0.36] | 0.31 ± 0.23* [0.23–0.39] | 0.32 ± 0.24* [0.24–0.41] | 0.38 ± 0.25* [0.30–0.46] | **0.64 ± 0.13† [0.56–0.72]** | <0.001 |
| F1 score | 0.50 ± 0.13* [0.44–0.56] | 0.47 ± 0.12* [0.40–0.53] | 0.39 ± 0.12* [0.32–0.45] | 0.40 ± 0.11* [0.33–0.46] | 0.17 ± 0.06* [0.10–0.23] | 0.16 ± 0.07* [0.10–0.23] | 0.26 ± 0.17* [0.19–0.32] | 0.24 ± 0.18* [0.18–0.31] | 0.26 ± 0.17*  [0.20–0.32] | 0.25 ± 0.17* [0.19–0.32] | **0.72 ± 0.10† [0.66–0.78]** | <0.001 |
| LOGAVD | 0.40 ± 0.25* [0.26–0.54] | 1.00 ± 0.32* [0.85–1.14] | 0.39 ± 0.28* [0.25–0.53] | 0.99 ± 0.38* [0.85–1.14] | 0.78 ± 0.39* [0.64–0.93] | 0.77 ± 0.30* [0.63–0.91] | 0.23 ± 0.18  [0.09–0.37] | 0.26 ± 0.23  [0.12–0.40] | 0.38 ± 0.29* [0.24–0.52] | 0.52 ± 0.37* [0.37–0.66] | **0.14 ± 0.09† [0.00–0.29]** | <0.001 |
| ICC (A, 1) |  |  |  |  |  |  |  |  |  |  |  |  |
| Coefficient | 0.58  [-0.10–0.87] | 0.21  [-0.05–0.60] | 0.57  [-0.10–0.86] | 0.18  [-0.06–0.56] | 0.25  [-0.09–0.64] | 0.28  [-0.06–0.69] | 0.77  [0.47–  0.91] | 0.71  [0.33–  0.89] | 0.52  [-0.01–  0.81] | 0.37  [-0.11–0.73] | **0.93† [0.64–**  **0.98]** |  |
| p-value | 0.001 | 0.001 | 0.001 | 0.002 | 0.003 | 0.001 | 0.001 | 0.001 | 0.002 | 0.003 | 0.001 |  |

**Supplementary Table 3: Performance metrics across the automated approaches by data source.**

Values in the table are mean ± standard deviation [95% confidence interval] from linear mixed-effects models. The lower part of the table indicates intraclass correlation coefficients and their p-values. Numbers in parentheses indicate the thresholds used in the automated algorithms. The dagger indicates the best-performing algorithm. The asterisks on the remaining algorithms denote a significant difference from the best algorithm. The best performing approach for each metric is represented in bold.

|  | BIANCA -LOO  (0.9) | BIANCA -LOO  (0.99) | BIANCA -training  (0.9) | BIANCA -training  (0.99) | FreeSurfer | FreeSurfer -FAST | LPA  (0.4) | LPA  (0.5) | LPA  -FAST  (0.4) | LPA  -FAST  (0.5) | U-Net | p-value |
| --- | --- | --- | --- | --- | --- | --- | --- | --- | --- | --- | --- | --- |
| **C-BIG**  **(n=55)** |  |  |  |  |  |  |  |  |  |  |  |  |
| Dice | 0.49 ± 0.18 [0.45–0.53] | 0.37 ± 0.15*  [0.33–0.41] | 0.47 ± 0.19  [0.42–0.51] | 0.39 ± 0.15*  [0.34–0.43] | 0.29 ± 0.16*  [0.25–0.33] | 0.30 ± 0.16* [0.26–0.34] | 0.05 ± 0.10* [0.01–0.09] | 0.05 ± 0.10*  [0.00–0.09] | 0.12 ± 0.17* [0.07–0.16] | 0.12 ± 0.18* [0.08–0.16] | **0.52 ± 0.17†  [0.48–0.56]** | < 0.001 |
| Hausdorff | 8.82 ± 2.45 [7.91–9.74] | **8.49 ± 3.17†**  **[7.58–9.40]** | 9.28 ± 2.40  [8.09–9.93] | 8.75 ± 2.81 [7.55–9.40] | 8.70 ± 3.21 [7.79–9.61] | 8.87 ± 3.39  [7.95–9.78] | 11.55 ± 3.95* [10.64–12.47] | 10.81 ± 4.18* [9.89–11.72] | 11.20 ± 4.10* [10.29–12.11] | 10.51 ± 4.21* [9.60–11.42] | 8.74 ± 2.33 [7.82–9.65] | < 0.001 |
| Recall | 0.64 ± 0.21* [0.59–0.69] | 0.48 ± 0.20* [0.43–0.53] | 0.66 ± 0.22* [0.59–0.70] | 0.48 ± 0.20*  [0.41–0.52] | 0.28 ± 0.17* [0.23–0.33] | 0.28 ± 0.18*  [0.23–0.34] | 0.09 ± 0.08*  [0.04–0.14] | 0.07 ± 0.08* [0.02–0.12] | 0.25 ± 0.25*  [0.20–0.31] | 0.21 ± 0.22*  [0.16–0.26] | **0.77 ± 0.24†  [0.72–0.83]** | < 0.001 |
| Precision | 0.33 ± 0.18* [0.29–0.37] | 0.42 ± 0.22* [0.38–0.46] | 0.18 ± 0.12*  [0.13–0.22] | 0.26 ± 0.16*  [0.22–0.30] | 0.14 ± 0.08* [0.10–0.18] | 0.14 ± 0.09* [0.11–0.18] | 0.06 ± 0.05* [0.02–0.10] | 0.07 ± 0.06* [0.03–0.11] | 0.14 ± 0.16* [0.11–0.18] | 0.16 ± 0.18*  [0.12–0.20] | **0.52 ± 0.19†  [0.48–0.56]** | < 0.001 |
| F1 score | 0.40 ± 0.19*  [0.37–0.44] | 0.41 ± 0.19* [0.37–0.45] | 0.26 ± 0.15* [0.21–0.29] | 0.31 ± 0.16* [0.26–0.34] | 0.16 ± 0.07* [0.12–0.20] | 0.17 ± 0.08* [0.13–0.20] | 0.07 ± 0.05* [0.03–0.11] | 0.07 ± 0.06* [0.03–0.10] | 0.17 ± 0.17* [0.13–0.21] | 0.16 ± 0.17* [0.12–0.20] | **0.59 ± 0.20†**  **[0.56–0.63]** | < 0.001 |
| LOGAVD | 0.28 ± 0.23  [0.19–0.38] | 0.56 ± 0.32* [0.46–0.65] | 0.33 ± 0.24  [0.23–0.43] | 0.47 ± 0.32* [0.37–0.57] | 0.40 ± 0.31 [0.30–0.49] | 0.41 ± 0.37  [0.32–0.51] | 0.41 ± 0.34 [0.31–0.50] | 0.28 ± 0.26 [0.18–0.38] | 0.47 ± 0.58* [0.38–0.57] | 0.39 ± 0.54 [0.30–0.49] | **0.25 ± 0.20† [0.15–0.35]** | < 0.001 |
| ICC (A, 1) |  |  |  |  |  |  |  |  |  |  |  |  |
| Coefficient | 0.90  [0.84–0.94] | 0.73  [-0.02–0.91] | 0.87  [0.78–0.92] | 0.77  [0.17–0.91] | 0.79  [0.65–0.88] | 0.75  [0.61–0.85] | 0.82  [0.36–0.93] | 0.90  [0.84–0.94] | 0.65  [0.39–0.80] | 0.72  [0.56–0.83] | **0.93†  [0.77–0.97]** |  |
| p-value | < 0.001 | < 0.001 | < 0.001 | < 0.001 | < 0.001 | < 0.001 | < 0.001 | < 0.001 | < 0.001 | < 0.001 | < 0.001 |  |
| **PPMI**  **(n=140)** |  |  |  |  |  |  |  |  |  |  |  |  |
| Dice | 0.36 ± 0.19*  [0.33–0.39] | 0.22 ± 0.15*  [0.19–0.25] | 0.33 ± 0.19*  [0.30–0.36] | 0.21 ± 0.15* [0.17–0.24] | 0.15 ± 0.13* [0.12–0.17] | 0.15 ± 0.14*  [0.12–0.18] | 0.32 ± 0.20*  [0.29–0.35] | 0.31 ± 0.20*  [0.28–0.34] | 0.31 ± 0.19*  [0.28–0.34] | 0.30 ± 0.19*  [0.27–0.33] | **0.41 ± 0.21†**  **[0.38–0.44]** | < 0.001 |
| Hausdorff | 6.70 ± 2.86  [6.14–7.27] | **6.55 ± 3.40†**  **[5.92–7.05]** | 7.08 ± 3.02  [6.15–7.33] | 6.91 ± 3.58 [5.99–7.16] | 7.82 ± 3.60* [7.26–8.38] | 7.91 ± 3.87* [7.34–8.47] | 7.46 ± 3.57*  [6.90–8.03] | 6.87 ± 3.37  [6.31–7.43] | 7.19 ± 3.35* [6.63–7.76] | 6.80 ± 3.31  [6.15–7.28] | 6.70 ± 3.21  [6.13–7.26] | < 0.001 |
| Recall | 0.59 ± 0.26*  [0.55–0.64] | 0.41 ± 0.26*  [0.37–0.45] | 0.66 ± 0.26  [0.59–0.68] | 0.48 ± 0.26* [0.41–0.50] | 0.27 ± 0.21* [0.22–0.31] | 0.28 ± 0.22*  [0.23–0.32] | 0.50 ± 0.27*  [0.45–0.54] | 0.42 ± 0.27*  [0.38–0.46] | 0.47 ± 0.29*  [0.43–0.52] | 0.40 ± 0.28*  [0.36–0.44] | **0.67 ± 0.28†**  **[0.63–0.71]** | < 0.001 |
| Precision | 0.30 ± 0.24* [0.26–0.33] | 0.35 ± 0.26*  [0.32–0.39] | 0.19 ± 0.19*  [0.17–0.25] | 0.28 ± 0.23* [0.26–0.34] | 0.08 ± 0.07* [0.05–0.12] | 0.09 ± 0.07*  [0.05–0.12] | 0.27 ± 0.19*  [0.24–0.31] | 0.33 ± 0.23*  [0.30–0.37] | 0.27 ± 0.18*  [0.23–0.30] | 0.34 ± 0.24*  [0.31–0.38] | **0.57 ± 0.27†**  **[0.54–0.61]** | < 0.001 |
| F1 score | 0.32 ± 0.19*  [0.30–0.35] | 0.31 ± 0.18*  [0.28–0.33] | 0.25 ± 0.17* [0.22–0.28] | 0.29 ± 0.17* [0.26–0.32] | 0.11 ± 0.07* [0.08–0.14] | 0.11 ± 0.08* [0.08–0.14] | 0.29 ± 0.16*  [0.27–0.32] | 0.30 ± 0.16*  [0.27–0.33] | 0.29 ± 0.16*  [0.26–0.31] | 0.30 ± 0.18*  [0.27–0.33] | **0.55 ± 0.21†**  **[0.52–0.57]** | < 0.001 |
| LOGAVD | 0.36 ± 0.30  [0.29–0.43] | 0.58 ± 0.45*  [0.51–0.65] | 0.40 ± 0.34  [0.30–0.47] | 0.63 ± 0.44* [0.53–0.70] | 0.61 ± 0.53* [0.53–0.70] | 0.62 ± 0.60* [0.55–0.69] | 0.47 ± 0.41  [0.40–0.54] | 0.41 ± 0.38  [0.34–0.48] | 0.42 ± 0.36  [0.35–0.49] | 0.41 ± 0.36  [0.34–0.48] | **0.34 ± 0.36†**  **[0.27–0.41]** | < 0.001 |
| ICC (A, 1) |  |  |  |  |  |  |  |  |  |  |  |  |
| Coefficient | **0.82†  [0.75–0.87]** | 0.59  [-0.03–0.82] | 0.77  [0.67–0.84] | 0.57 [-0.02–0.81] | 0.51  [0.36–0.63] | 0.47  [0.31–0.60] | 0.75  [0.64–0.83] | 0.79  [0.72–0.85] | 0.77  [0.69–0.83] | 0.78  [0.69–0.84] | 0.81  [0.74–0.86] |  |
| p-value | < 0.001 | < 0.001 | < 0.001 | < 0.001 | < 0.001 | < 0.001 | < 0.001 | < 0.001 | < 0.001 | < 0.001 | < 0.001 |  |
| **UPenn**  **(n=70)** |  |  |  |  |  |  |  |  |  |  |  |  |
| Dice | 0.43 ± 0.20  [0.39–0.48] | 0.28 ± 0.14*  [0.24–0.33] | 0.41 ± 0.21*  [0.35–0.44] | 0.27 ± 0.14* [0.21–0.31] | 0.22 ± 0.17* [0.17–0.27] | 0.23 ± 0.17* [0.18–0.27] | 0.40 ± 0.22*  [0.35–0.44] | 0.37 ± 0.22*  [0.32–0.41] | 0.39 ± 0.20*  [0.34–0.43] | 0.34 ± 0.19*  [0.29–0.39] | **0.46 ± 0.22†**  **[0.42–0.51]** | < 0.001 |
| Hausdorff | 11.86 ± 6.06 [10.38–13.34] | 12.19 ± 7.52*  [10.71–13.67] | 13.45 ± 6.10*  [11.41–14.40] | 13.21 ± 8.03* [11.18–14.17] | 13.89 ± 6.22*  [12.41–15.38] | 13.56 ± 6.00* [12.08–15.05] | 10.94 ± 5.11  [9.46–12.42] | **10.83 ± 5.50† [9.35–12.31]** | 11.32 ± 6.42  [9.84–12.80] | 11.42 ± 7.01  [9.94–12.91] | 11.06 ± 4.42  [9.58–12.54] | < 0.001 |
| Recall | 0.61 ± 0.21*  [0.56–0.66] | 0.46 ± 0.23*  [0.41–0.51] | 0.62 ± 0.21*  [0.57–0.68] | 0.45 ± 0.22* [0.40–0.50] | 0.27 ± 0.23* [0.22–0.32] | 0.26 ± 0.24* [0.21–0.31] | 0.31 ± 0.21*  [0.26–0.36] | 0.26 ± 0.21*  [0.21–0.31] | 0.30 ± 0.20*  [0.25–0.35] | 0.24 ± 0.19*  [0.19–0.29] | **0.73 ± 0.20†**  **[0.68–0.78]** | < 0.001 |
| Precision | 0.26 ± 0.19* [0.22–0.31] | 0.37 ± 0.22*  [0.33–0.42] | 0.13 ± 0.11*  [0.09–0.18] | 0.21 ± 0.16* [0.17–0.26] | 0.09 ± 0.08* [0.04–0.13] | 0.08 ± 0.08* [0.04–0.13] | 0.38 ± 0.21*  [0.34–0.43] | 0.41 ± 0.22*  [0.37–0.46] | 0.45 ± 0.21  [0.40–0.49] | **0.48 ± 0.21†**  **[0.44–0.52]** | 0.40 ± 0.21*  [0.35–0.44] | < 0.001 |
| F1 score | 0.32 ± 0.18*  [0.29–0.35] | 0.35 ± 0.17*  [0.32–0.39] | 0.19 ± 0.14*  [0.16–0.23] | 0.25 ± 0.15* [0.21–0.29] | 0.10 ± 0.06* [0.07–0.14] | 0.10 ± 0.06* [0.06–0.13] | 0.29 ± 0.13*  [0.26–0.32] | 0.27 ± 0.14*  [0.24–0.30] | 0.31 ± 0.14*  [0.28–0.35] | 0.28 ± 0.14*  [0.25–0.32] | **0.47 ± 0.20†**  **[0.44–0.51]** | < 0.001 |
| LOGAVD | 0.31 ± 0.27  [0.23–0.38] | 0.57 ± 0.43*  [0.49–0.65] | 0.36 ± 0.32  [0.27–0.43] | 0.59 ± 0.46* [0.50–0.66] | 0.52 ± 0.38* [0.45–0.60] | 0.49 ± 0.33* [0.42–0.57] | 0.31 ± 0.23  [0.23–0.38] | 0.36 ± 0.28  [0.28–0.43] | 0.36 ± 0.29  [0.28–0.44] | 0.43 ± 0.36*  [0.36–0.51] | **0.27 ± 0.22†**  **[0.19–0.35]** | < 0.001 |
| ICC (A, 1) |  |  |  |  |  |  |  |  |  |  |  |  |
| Coefficient | 0.91  [0.86–0.94] | 0.73 [0.04–0.90] | 0.87  [0.78–0.92] | 0.70  [0.05–0.89] | 0.72  [0.59–0.82] | 0.77  [0.65–0.85] | 0.92  [0.87–0.95] | 0.90  [0.67–0.96] | 0.88  [0.70–0.94] | 0.83  [0.34–0.93] | **0.93†  [0.85–0.97]** |  |
| p-value | < 0.001 | < 0.001 | < 0.001 | < 0.001 | < 0.001 | < 0.001 | < 0.001 | < 0.001 | < 0.001 | < 0.001 | < 0.001 |  |

**Supplementary Table 4: Performance metrics across the automated approaches by magnetic field strength.**

Values in the table are mean ± standard deviation [95% confidence interval] from linear mixed-effects models. The lower part of the table indicates intraclass correlation coefficients and their p-values. Numbers in parentheses indicate the thresholds used in the automated algorithms. The dagger indicates the best-performing algorithm. The asterisks on the remaining algorithms denote a significant difference from the best algorithm. The best performing approach for each metric is represented in bold.

|  | BIANCA -LOO  (0.9) | BIANCA -LOO  (0.99) | BIANCA -training (0.9) | BIANCA -training (0.99) | FreeSurfer | FreeSurfer -FAST | LPA  (0.4) | LPA  (0.5) | LPA  -FAST  (0.4) | LPA  -FAST  (0.5) | U-Net | p-value |
| --- | --- | --- | --- | --- | --- | --- | --- | --- | --- | --- | --- | --- |
| **1.5T**  **(n=88)** |  |  |  |  |  |  |  |  |  |  |  |  |
| Dice | 0.35 ± 0.20* [0.31–0.39] | 0.22 ± 0.16* [0.18–0.26] | 0.33 ± 0.19* [0.28–0.36] | 0.22 ± 0.17* [0.17–0.25] | 0.14 ± 0.13* [0.10–0.17] | 0.15 ± 0.14* [0.11–0.19] | 0.30 ± 0.20* [0.26–0.34] | 0.30 ± 0.21* [0.26–0.34] | 0.32 ± 0.20* [0.28–0.35] | 0.31 ± 0.20* [0.27–0.35] | **0.41 ± 0.21† [0.37–0.44]** | < 0.001 |
| Hausdorff | 5.96 ± 2.38 [5.40–6.51] | **5.71 ± 2.88† [5.08–6.20]** | 6.51 ± 2.53 [5.47–6.62] | 6.19 ± 3.11 [5.15–6.30] | 6.76 ± 2.74* [6.21–7.32] | 6.67 ± 2.58* [6.11–7.22] | 6.47 ± 2.78* [5.91–7.02] | 5.87 ± 2.59 [5.31–6.43] | 6.15 ± 2.48* [5.60–6.71] | 5.78 ± 2.44 [5.11–6.23] | 5.89 ± 2.60 [5.34–6.45] | < 0.001 |
| Recall | 0.60 ± 0.29* [0.54–0.65] | 0.42 ± 0.28* [0.37–0.48] | 0.69 ± 0.27 [0.61–0.73] | 0.49 ± 0.29* [0.41–0.54] | 0.28 ± 0.23* [0.22–0.34] | 0.29 ± 0.24* [0.23–0.35] | 0.50 ± 0.28* [0.44–0.56] | 0.42 ± 0.29* [0.36–0.48] | 0.49 ± 0.31* [0.43–0.55] | 0.42 ± 0.30* [0.36–0.48] | **0.69 ± 0.28† [0.63–0.74]** | < 0.001 |
| Precision | 0.31 ± 0.25* [0.26–0.35] | 0.37 ± 0.28* [0.32–0.41] | 0.22 ± 0.21* [0.18–0.28] | 0.31 ± 0.25* [0.27–0.37] | 0.08 ± 0.07* [0.03–0.13] | 0.08 ± 0.06* [0.03–0.13] | 0.29 ± 0.20* [0.24–0.33] | 0.36 ± 0.24* [0.31–0.40] | 0.29 ± 0.18* [0.24–0.33] | 0.37 ± 0.26* [0.33–0.42] | **0.56 ± 0.28† [0.51–0.60]** | < 0.001 |
| F1 score | 0.33 ± 0.20* [0.29–0.36] | 0.31 ± 0.19* [0.28–0.35] | 0.26 ± 0.18* [0.23–0.31] | 0.30 ± 0.17* [0.26–0.34] | 0.11 ± 0.08* [0.07–0.14] | 0.11 ± 0.07* [0.07–0.14] | 0.30 ± 0.16* [0.27–0.34] | 0.30 ± 0.17* [0.27–0.34] | 0.30 ± 0.16* [0.26–0.34] | 0.32 ± 0.20* [0.28–0.35] | **0.54 ± 0.22† [0.51–0.58]** | < 0.001 |
| LOGAVD | 0.38 ± 0.31 [0.30–0.46] | 0.54 ± 0.45* [0.47–0.62] | 0.44 ± 0.34 [0.31–0.49] | 0.61 ± 0.47* [0.48–0.66] | 0.53 ± 0.32* [0.46–0.61] | 0.52 ± 0.32* [0.44–0.59] | 0.47 ± 0.40* [0.39–0.55] | 0.42 ± 0.38 [0.34–0.50] | 0.41 ± 0.34 [0.33–0.49] | 0.40 ± 0.35 [0.32–0.47] | **0.33 ± 0.34† [0.25–0.40]** | < 0.001 |
| *ICC (A, 1)* |  |  |  |  |  |  |  |  |  |  |  |  |
| Coefficient | 0.80  [0.72–0.87] | 0.62  [0.03–0.83] | 0.76  [0.64–0.85] | 0.59  [0.01–0.82] | 0.63  [0.46–0.75] | 0.65  [0.47–0.77] | 0.76  [0.63–0.84] | 0.78  [0.69–0.85] | 0.79  [0.70–0.86] | 0.79  [0.68–0.86] | **0.83† [0.75–0.88]** |  |
| p-value | < 0.001 | < 0.001 | < 0.001 | < 0.001 | < 0.001 | < 0.001 | < 0.001 | < 0.001 | < 0.001 | < 0.001 | < 0.001 |  |
| **3T**  **(n=177)** |  |  |  |  |  |  |  |  |  |  |  |  |
| Dice | 0.43 ± 0.19 [0.40–0.46] | 0.29 ± 0.15* [0.26–0.32] | 0.41 ± 0.20* [0.38–0.44] | 0.30 ± 0.16* [0.26–0.32] | 0.22 ± 0.16* [0.20–0.25] | 0.23 ± 0.17* [0.20–0.26] | 0.27 ± 0.24* [0.24–0.30] | 0.26 ± 0.23* [0.23–0.29] | 0.28 ± 0.21* [0.25–0.31] | 0.26 ± 0.20* [0.23–0.29] | **0.47 ± 0.20† [0.44–0.50]** | < 0.001 |
| Hausdorff | 9.77 ± 4.71 [9.01–10.53] | 9.79 ± 5.76 [9.03–10.54] | 10.68 ± 5.05*  [9.48–11.03] | 10.42 ± 6.16 [9.23–10.77] | 11.02 ± 5.38*  [10.27–11.78] | 11.06 ± 5.33* [10.30–11.82] | 10.61 ± 4.57*  [9.85–11.36] | 10.16 ± 4.76*  [9.40–10.91] | 10.59 ± 5.18*  [9.83–11.34] | 10.27 ± 5.52*  [9.52–11.03] | **9.45 ± 3.88† [8.70–10.21]** | < 0.001 |
| Recall | 0.62 ± 0.21* [0.58–0.65] | 0.44 ± 0.22* [0.41–0.48] | 0.64 ± 0.22* [0.59–0.66] | 0.46 ± 0.20* [0.42–0.49] | 0.26 ± 0.20*  [0.23–0.30] | 0.27 ± 0.21* [0.23–0.30] | 0.30 ± 0.25* [0.26–0.33] | 0.25 ± 0.23* [0.22–0.28] | 0.33 ± 0.24* [0.29–0.36] | 0.27 ± 0.22* [0.23–0.30] | **0.72 ± 0.24† [0.68–0.75]** | < 0.001 |
| Precision | 0.29 ± 0.20* [0.26–0.32] | 0.37 ± 0.22* [0.34–0.40] | 0.15 ± 0.12* [0.13–0.19] | 0.23 ± 0.17* [0.21–0.27] | 0.10 ± 0.08* [0.08–0.13] | 0.10 ± 0.09* [0.08–0.13] | 0.24 ± 0.21* [0.22–0.27] | 0.27 ± 0.23* [0.24–0.30] | 0.29 ± 0.22* [0.26–0.32] | 0.33 ± 0.24* [0.30–0.35] | **0.49 ± 0.23† [0.46–0.52]** | < 0.001 |
| F1 score | 0.34 ± 0.18* [0.32–0.37] | 0.35 ± 0.18* [0.33–0.38] | 0.22 ± 0.15* [0.20–0.25] | 0.28 ± 0.16* [0.25–0.30] | 0.12 ± 0.07* [0.10–0.15] | 0.12 ± 0.08* [0.10–0.15] | 0.22 ± 0.16* [0.19–0.24] | 0.21 ± 0.16* [0.19–0.24] | 0.25 ± 0.16* [0.23–0.28] | 0.24 ± 0.16* [0.22–0.26] | **0.53 ± 0.21† [0.51–0.56]** | < 0.001 |
| LOGAVD | 0.31 ± 0.26 [0.25–0.37] | 0.59 ± 0.40* [0.53–0.65] | 0.34 ± 0.30 [0.28–0.41] | 0.57 ± 0.40* [0.51–0.64] | 0.55 ± 0.51* [0.49–0.60] | 0.55 ± 0.57* [0.50–0.61] | 0.38 ± 0.34 [0.32–0.44] | 0.34 ± 0.31 [0.28–0.40] | 0.42 ± 0.43* [0.36–0.48] | 0.42 ± 0.42* [0.36–0.48] | **0.29 ± 0.29†**  **[0.23–0.35]** | < 0.001 |
| *ICC (A, 1)* |  |  |  |  |  |  |  |  |  |  |  |  |
| Coefficient | 0.89  [0.86–0.92] | 0.70  [-0.01–0.89] | 0.86  [0.81–0.90] | 0.70  [0.07–0.88] | 0.62 [0.52–0.70] | 0.59  [0.48–0.68] | 0.85  [0.80–0.89] | 0.88  [0.84–0.91] | 0.79  [0.72–0.84] | 0.79  [0.71–0.84] | **0.90† [0.85–0.93]** |  |
| p-value | < 0.001 | < 0.001 | < 0.001 | < 0.001 | < 0.001 | < 0.001 | < 0.001 | < 0.001 | < 0.001 | < 0.001 | < 0.001 |  |

**Supplementary Table 5: Performance metrics across the automated approaches by FLAIR acquisition dimension**

Values in the table are mean ± standard deviation [95% confidence interval] from linear mixed-effects models. The lower part of the table indicates intraclass correlation coefficients and their p-values. Numbers in parentheses indicate the thresholds used in the automated algorithms. The dagger indicates the best-performing algorithm. The asterisks on the remaining algorithms denote a significant difference from the best algorithm. The best performing approach for each metric is represented in bold.

|  | BIANCA -LOO  (0.9) | BIANCA -LOO  (0.99) | BIANCA -training  (0.9) | BIANCA -training  (0.99) | FreeSurfer | FreeSurfer -FAST | LPA  (0.4) | LPA  (0.5) | LPA  -FAST  (0.4) | LPA  -FAST  (0.5) | U-Net | p-value |
| --- | --- | --- | --- | --- | --- | --- | --- | --- | --- | --- | --- | --- |
| **2D**  **(n=195)** |  |  |  |  |  |  |  |  |  |  |  |  |
| Dice | 0.39 ± 0.20*  [0.37–0.42] | 0.26 ± 0.16*  [0.24–0.29] | 0.38 ± 0.20*  [0.35–0.40] | 0.27 ± 0.18*  [0.24–0.30] | 0.19 ± 0.16*  [0.16–0.21] | 0.20 ± 0.16* [0.17–0.22[ | 0.24 ± 0.21* [0.21–0.27] | 0.24 ± 0.22* [0.21–0.26] | 0.26 ± 0.20* [0.23–0.29] | 0.25 ± 0.20* [0.22–0.28] | **0.44 ± 0.20† [0.41–0.47]** | < 0.001 |
| Hausdorff | 7.30 ± 2.91  [6.79–7.81] | **7.10 ± 3.44† [6.54–7.56]** | 7.82 ± 3.00  [6.84–7.89] | 7.52 ± 3.44  [6.55–7.60] | 8.07 ± 3.51*  [7.56–8.58] | 8.18 ± 3.76* [7.67–8.69] | 8.62 ± 4.11* [8.11–9.13] | 7.98 ± 4.02* [7.47–8.49] | 8.32 ± 4.00* [7.81–8.83] | 7.86 ± 3.95* [7.28–8.30] | 7.27 ± 3.12 [6.76–7.78] | < 0.001 |
| Recall | 0.61 ± 0.25*  [0.57–0.64] | 0.43 ± 0.24* [0.39–0.47] | 0.66 ± 0.25  [0.60–0.68] | 0.48 ± 0.24*  [0.42–0.50] | 0.27 ± 0.20*  [0.23–0.31] | 0.28 ± 0.21* [0.24–0.31] | 0.38 ± 0.30* [0.34–0.42] | 0.32 ± 0.28* [0.29–0.36] | 0.41 ± 0.29* [0.38–0.45] | 0.35 ± 0.28* [0.31–0.38] | **0.70 ± 0.27† [0.66–0.74]** | < 0.001 |
| Precision | 0.31 ± 0.22*  [0.28–0.34] | 0.37 ± 0.25* [0.34–0.40] | 0.19 ± 0.17*  [0.17–0.23] | 0.27 ± 0.21*  [0.26–0.32] | 0.10 ± 0.08*  [0.07–0.13] | 0.10 ± 0.08* [0.07–0.13] | 0.21 ± 0.19* [0.19–0.24] | 0.26 ± 0.23* [0.23–0.29] | 0.23 ± 0.18* [0.21–0.26] | 0.29 ± 0.24* [0.26–0.32] | **0.56 ± 0.25† [0.53–0.59]** | < 0.001 |
| F1 score | 0.35 ± 0.19*  [0.32–0.37] | 0.34 ± 0.19* [0.31–0.36] | 0.25 ± 0.17*  [0.23–0.28] | 0.30 ± 0.17*  [0.27–0.32] | 0.12 ± 0.08*  [0.10–0.15] | 0.13 ± 0.08* [0.10–0.15] | 0.23 ± 0.17* [0.21–0.25] | 0.23 ± 0.18* [0.21–0.26] | 0.25 ± 0.17* [0.23–0.28] | 0.26 ± 0.19* [0.24–0.28] | **0.56 ± 0.21† [0.54–0.58]** | < 0.001 |
| LOGAVD | 0.34 ± 0.28  [0.28–0.40] | 0.58 ± 0.42* [0.52–0.63] | 0.38 ± 0.31  [0.31–0.43] | 0.58 ± 0.41*  [0.51–0.64] | 0.55 ± 0.48*  [0.49–0.60] | 0.56 ± 0.55* [0.50–0.62] | 0.45 ± 0.39* [0.39–0.51] | 0.37 ± 0.35 [0.32–0.43] | 0.44 ± 0.43* [0.38–0.49] | 0.40 ± 0.41 [0.35–0.46] | **0.32 ± 0.33† [0.26–0.37]** | < 0.001 |
| *ICC (A, 1)* |  |  |  |  |  |  |  |  |  |  |  |  |
| Coefficient | 0.85  [0.81–0.89] | 0.66  [-0.01–0.86] | 0.82  [0.76–0.87] | 0.67  [0.06–0.86] | 0.59  [0.49–0.67] | 0.55  [0.44–0.64] | 0.79  [0.65–0.86] | 0.84  [0.79–0.87] | 0.76  [0.68–0.82] | 0.78  [0.72–0.83] | **0.86† [0.81–0.89]** |  |
| p-value | < 0.001 | < 0.001 | < 0.001 | < 0.001 | < 0.001 | < 0.001 | < 0.001 | < 0.001 | < 0.001 | < 0.001 | < 0.001 |  |
| **3D  (n=70)** |  |  |  |  |  |  |  |  |  |  |  |  |
| Dice | 0.43 ± 0.20  [0.39–0.48] | 0.28 ± 0.14*  [0.24–0.33] | 0.41 ± 0.21*  [0.35–0.44] | 0.27 ± 0.14*  [0.21–0.31] | 0.22 ± 0.17*  [0.17–0.27] | 0.23 ± 0.17* [0.18–0.27] | 0.40 ± 0.22* [0.35–0.44] | 0.37 ± 0.22* [0.32–0.41] | 0.39 ± 0.20* [0.34–0.43] | 0.34 ± 0.19* [0.29–0.39] | **0.46 ± 0.22† [0.42–0.51]** | < 0.001 |
| Hausdorff | 11.86 ± 6.06*  [10.38–13.34] | 12.19 ± 7.52*  [10.71–13.67] | 13.45 ± 6.10*  [11.41–14.40] | 13.21 ± 8.03* [11.18–14.17] | 13.89 ± 6.22*  [12.41–15.38] | 13.56 ± 6.00*  [12.08–15.05] | 10.94 ± 5.11 [9.46–12.42] | **10.83 ± 5.50† [9.35–12.31]** | 11.32 ± 6.42 [9.84–12.80] | 11.42 ± 7.01 [9.94–12.91] | 11.06 ± 4.42 [9.58–12.54] | < 0.001 |
| Recall | 0.61 ± 0.21*  [0.56–0.66] | 0.46 ± 0.23*  [0.41–0.51] | 0.62 ± 0.21*  [0.57–0.68] | 0.45 ± 0.22*  [0.40–0.50] | 0.27 ± 0.23*  [0.22–0.32] | 0.26 ± 0.24* [0.21–0.31] | 0.31 ± 0.21* [0.26–0.36] | 0.26 ± 0.21* [0.21–0.31] | 0.30 ± 0.20* [0.25–0.35] | 0.24 ± 0.19* [0.19–0.29] | **0.73 ± 0.20† [0.68–0.78]** | < 0.001 |
| Precision | 0.26 ± 0.19* [0.22–0.31] | 0.37 ± 0.22  [0.33–0.42] | 0.13 ± 0.11*  [0.09–0.18] | 0.21 ± 0.16*  [0.17–0.26] | 0.09 ± 0.08*  [0.04–0.13] | 0.08 ± 0.08* [0.04–0.13] | 0.38 ± 0.21 [0.34–0.43] | 0.41 ± 0.22 [0.37–0.46] | 0.45 ± 0.21 [0.40–0.49] | **0.48 ± 0.21† [0.44–0.52]** | 0.40 ± 0.21 [0.35–0.44] | < 0.001 |
| F1 score | 0.32 ± 0.18*  [0.29–0.35] | 0.35 ± 0.17*  [0.32–0.39] | 0.19 ± 0.14*  [0.16–0.23] | 0.25 ± 0.15*  [0.21–0.29] | 0.10 ± 0.06*  [0.07–0.14] | 0.10 ± 0.06* [0.06–0.13] | 0.29 ± 0.13* [0.26–0.32] | 0.27 ± 0.14* [0.24–0.30] | 0.31 ± 0.14* [0.28–0.35] | 0.28 ± 0.14* [0.25–0.32] | **0.47 ± 0.20† [0.44–0.51]** | < 0.001 |
| LOGAVD | 0.31 ± 0.27  [0.23–0.38] | 0.57 ± 0.43*  [0.49–0.65] | 0.36 ± 0.32  [0.26–0.43] | 0.59 ± 0.46*  [0.50–0.66] | 0.52 ± 0.38*  [0.45–0.60] | 0.49 ± 0.33* [0.42–0.57] | 0.31 ± 0.23 [0.23–0.38] | 0.36 ± 0.28 [0.28–0.43] | 0.36 ± 0.29 [0.28–0.44] | 0.43 ± 0.36* [0.36–0.51] | **0.27 ± 0.22† [0.19–0.35]** | < 0.001 |
| *ICC (A, 1)* |  |  |  |  |  |  |  |  |  |  |  |  |
| Coefficient | 0.91  [0.86–0.94] | 0.73  [0.04–0.90] | 0.87  [0.78–0.92] | 0.70  [0.05–0.89] | 0.72  [0.59–0.82] | 0.77  [0.65–0.85] | 0.92  [0.87–0.95] | 0.90  [0.67–0.96] | 0.88  [0.70–0.94] | 0.83  [0.34–0.93] | **0.93† [0.85–0.97]** |  |
| p-value | < 0.001 | < 0.001 | < 0.001 | < 0.001 | < 0.001 | < 0.001 | < 0.001 | < 0.001 | < 0.001 | < 0.001 | < 0.001 |  |

**Supplementary Table 6: Performance metrics across the automated approaches by MRI manufacturer**

Values in the table are mean ± standard deviation [95% confidence interval] from linear mixed-effects models. The lower part of the table indicates intraclass correlation coefficients and their p-values. Numbers in parentheses indicate the thresholds used in the automated algorithms. The dagger indicates the best-performing algorithm. The asterisks on the remaining algorithms denote a significant difference from the best algorithm. The best performing approach for each metric is represented in bold.

|  | BIANCA -LOO  (0.9) | BIANCA -LOO  (0.99) | BIANCA -training  (0.9) | BIANCA -training  (0.99) | FreeSurfer | FreeSurfer -FAST | LPA  (0.4) | LPA  (0.5) | LPA  -FAST  (0.4) | LPA  -FAST  (0.5) | U-Net | p-value |
| --- | --- | --- | --- | --- | --- | --- | --- | --- | --- | --- | --- | --- |
| **GE**  **(n=80)** |  |  |  |  |  |  |  |  |  |  |  |  |
| Dice | 0.36 ± 0.19  [0.32–0.40] | 0.23 ± 0.15*  [0.19–0.27] | 0.33 ± 0.19* [0.29–0.37] | 0.21 ± 0.16* [0.17–0.26] | 0.14 ± 0.14*  [0.10–0.18] | 0.15 ± 0.15*  [0.11–0.19] | 0.33 ± 0.20*  [0.29–0.37] | 0.32 ± 0.20*  [0.28–0.36] | 0.32 ± 0.18*  [0.28–0.36] | 0.31 ± 0.19*  [0.27–0.35] | **0.40 ± 0.21†**  **[0.36–0.44]** | <0.001 |
| Hausdorff | 6.90 ± 2.74  [6.17–7.63] | **6.66 ± 3.35† [5.93–7.39]** | 7.03 ± 2.93 [6.22–7.74] | 6.81 ± 3.45 [5.99–7.52] | 8.21 ± 3.61*  [7.48–8.94] | 8.32 ± 3.95*  [7.59–9.05] | 7.32 ± 3.02  [6.59–8.04] | 6.83 ± 3.01  [6.10–7.56] | 7.51 ± 3.27*  [6.78–8.24] | 6.95 ± 3.32  [6.23–7.68] | 6.92 ± 3.41  [6.19–7.65] | <0.001 |
| Recall | 0.59 ± 0.25  [0.53–0.64] | 0.40 ± 0.25*  [0.34–0.46] | 0.64 ± 0.27 [0.55–0.67] | 0.46 ± 0.25* [0.37–0.50] | 0.27 ± 0.21*  [0.21–0.32] | 0.27 ± 0.23* [0.21–0.33] | 0.50 ± 0.29*  [0.44–0.56] | 0.43 ± 0.29*  [0.37–0.49] | 0.49 ± 0.30*  [0.43–0.55] | 0.41 ± 0.29*  [0.35–0.47] | **0.66 ± 0.28† [0.60–0.71]** | <0.001 |
| Precision | 0.29 ± 0.23*  [0.25–0.34] | 0.35 ± 0.24*  [0.30–0.39] | 0.20 ± 0.21*  [0.17–0.26] | 0.28 ± 0.23* [0.24–0.34] | 0.07 ± 0.06*  [0.03–0.12] | 0.08 ± 0.07* [0.03–0.12] | 0.28 ± 0.18*  [0.23–0.32] | 0.34 ± 0.23*  [0.30–0.39] | 0.26 ± 0.17* [0.21–0.30] | 0.32 ± 0.23*  [0.28–0.37] | **0.58 ± 0.28†**  **[0.53–0.62]** | <0.001 |
| F1 score | 0.32 ± 0.19*  [0.28–0.35] | 0.30 ± 0.17*  [0.27–0.34] | 0.24 ± 0.19* [0.21–0.28] | 0.27 ± 0.18* [0.24–0.32] | 0.10 ± 0.07*  [0.06–0.13] | 0.10 ± 0.08*  [0.06–0.13] | 0.29 ± 0.15*  [0.26–0.33] | 0.30 ± 0.17*  [0.27–0.34] | 0.28 ± 0.15*  [0.24–0.32] | 0.29 ± 0.16*  [0.25–0.32] | **0.54 ± 0.22†**  **[0.50–0.57]** | <0.001 |
| LOGAVD | 0.40 ± 0.30  [0.30–0.50] | 0.60 ± 0.45*  [0.50–0.70] | 0.44 ± 0.36 [0.31–0.55] | 0.62 ± 0.43* [0.49–0.73] | 0.66 ± 0.64*  [0.56–0.76] | 0.69 ± 0.72*  [0.59–0.79] | 0.44 ± 0.37  [0.34–0.54] | 0.41 ± 0.35  [0.31–0.51] | 0.44 ± 0.38  [0.34–0.54] | 0.41 ± 0.36  [0.31–0.51] | **0.37 ± 0.43†**  **[0.27–0.47]** | 0.006 |
| ICC (A, 1) |  |  |  |  |  |  |  |  |  |  |  |  |
| Coefficient | 0.80 [0.71–0.87] | 0.61 [-0.02–0.84] | 0.73 [0.57–0.83] | 0.58 [0.01–0.81] | 0.46 [0.25–0.62] | 0.40 [0.19–0.58] | 0.78 [0.68–0.86] | **0.81 [0.72–0.87]** | 0.76 [0.65–0.84] | 0.79 [0.68–0.86] | 0.77 [0.66–0.85] |  |
| p-value | < 0.001 | < 0.001 | < 0.001 | < 0.001 | < 0.001 | < 0.001 | < 0.001 | < 0.001 | < 0.001 | < 0.001 | < 0.001 |  |
| **Philips**  **(n=29)** |  |  |  |  |  |  |  |  |  |  |  |  |
| Dice | 0.34 ± 0.21*  [0.27–0.40] | 0.18 ± 0.16*  [0.12–0.25] | 0.34 ± 0.19*  [0.25–0.39] | 0.19 ± 0.13* [0.10–0.24] | 0.14 ± 0.12* [0.08–0.20] | 0.15 ± 0.12* [0.09–0.22] | 0.26 ± 0.17* [0.19–0.32] | 0.26 ± 0.19* [0.20–0.33] | 0.32 ± 0.20* [0.26–0.38] | 0.31 ± 0.20* [0.24–0.37] | **0.44 ± 0.16†  [0.38–0.51]** | <0.001 |
| Hausdorff | **6.13 ± 3.52† [4.59–7.68]** | 6.45 ± 3.79 [4.63–7.74] | 7.32 ± 3.80 [4.68–7.89] | 7.22 ± 4.26 [4.57–7.79] | 7.33 ± 4.27  [5.79–8.88] | 7.35 ± 4.60  [5.81–8.90] | 8.22 ± 4.93* [6.68–9.77] | 7.40 ± 4.47 [5.85–8.94] | 6.79 ± 4.20  [5.24–8.34] | 6.88 ± 3.95 [4.87–7.98] | 6.20 ± 3.34 [4.66–7.75] | <0.001 |
| Recall | 0.55 ± 0.30*  [0.44–0.65] | 0.35 ± 0.28*  [0.25–0.45] | 0.66 ± 0.22 [0.52–0.75] | 0.47 ± 0.28* [0.34–0.56] | 0.27 ± 0.22* [0.16–0.37] | 0.28 ± 0.23* [0.18–0.39] | 0.50 ± 0.29* [0.39–0.60] | 0.42 ± 0.27* [0.32–0.53] | 0.47 ± 0.31* [0.37–0.57] | 0.42 ± 0.31* [0.31–0.52] | **0.72 ± 0.25†  [0.62–0.82]** | <0.001 |
| Precision | 0.39 ± 0.31* [0.29–0.49] | 0.42 ± 0.36*  [0.32–0.51] | 0.26 ± 0.20* [0.17–0.39] | 0.37 ± 0.30* [0.28–0.50] | 0.09 ± 0.08* [-0.01–0.19] | 0.09 ± 0.07* [-0.00–0.19] | 0.26 ± 0.25* [0.16–0.36] | 0.32 ± 0.27* [0.22–0.42] | 0.29 ± 0.23* [0.20–0.39] | 0.41 ± 0.35* [0.31–0.50] | **0.61 ± 0.28† [0.51–0.71]** | <0.001 |
| F1 score | 0.36 ± 0.24* [0.29–0.43] | 0.30 ± 0.22*  [0.23–0.37] | 0.31 ± 0.16* [0.23–0.39] | 0.32 ± 0.17* [0.24–0.40] | 0.11 ± 0.07* [0.04–0.18] | 0.12 ± 0.07* [0.05–0.19] | 0.25 ± 0.17* [0.18–0.32] | 0.27 ± 0.16* [0.20–0.33] | 0.28 ± 0.20* [0.22–0.35] | 0.31 ± 0.25* [0.24–0.38] | **0.59 ± 0.21† [0.52–0.65]** | <0.001 |
| LOGAVD | 0.38 ± 0.35  [0.23–0.53] | 0.63 ± 0.49* [0.48–0.78] | 0.45 ± 0.37 [0.24–0.60] | 0.75 ± 0.48* [0.54–0.90] | 0.59 ± 0.36  [0.44–0.74] | 0.55 ± 0.36  [0.39–0.70] | 0.66 ± 0.51* [0.51–0.81] | 0.51 ± 0.49 [0.36–0.66] | 0.46 ± 0.39 [0.31–0.61] | 0.45 ± 0.39 [0.29–0.60] | **0.31 ± 0.25† [0.16–0.46]** | <0.001 |
| ICC (A, 1) |  |  |  |  |  |  |  |  |  |  |  |  |
| Coefficient | 0.73 [0.46–0.87] | 0.45 [-0.09–0.76] | 0.68 [0.34–0.86] | 0.40 [-0.10–0.74] | 0.56 [0.26–0.77] | 0.60 [0.31–0.79] | 0.59 [0.25–0.79] | 0.65 [0.39–0.82] | 0.71 [0.47–0.85] | 0.71  [0.47–0.85] | **0.85† [0.71–0.93]** |  |
| p-value | < 0.001 | < 0.001 | < 0.001 | 0.003 | < 0.001 | < 0.001 | < 0.001 | < 0.001 | < 0.001 | < 0.001 | < 0.001 |  |
| **Siemens**  **(n=156)** |  |  |  |  |  |  |  |  |  |  |  |  |
| Dice | 0.44 ± 0.20  [0.41–0.47] | 0.30 ± 0.15*  [0.27–0.34] | 0.42 ± 0.21* [0.38–0.44] | 0.31 ± 0.16* [0.27–0.33] | 0.23 ± 0.17*  [0.20–0.26] | 0.24 ± 0.17*  [0.21–0.27] | 0.26 ± 0.24*  [0.23–0.29] | 0.25 ± 0.24*  [0.21–0.28] | 0.27 ± 0.22*  [0.24–0.30] | 0.25 ± 0.21*  [0.22–0.28] | **0.47 ± 0.21†**  **[0.44–0.51]** | <0.001 |
| Hausdorff | 9.77 ± 4.88  [8.93–10.60] | 9.72 ± 6.03  [8.89–10.55] | 10.73 ± 5.13* [9.41–11.09] | 10.41 ± 6.34 [9.08–10.77] | 10.75 ± 5.54*  [9.91–11.58] | 10.68 ± 5.35*  [9.85–11.51] | 10.40 ± 4.70*  [9.57–11.24] | 9.96 ± 4.95  [9.12–10.79] | 10.37 ± 5.36*  [9.54–11.20] | 10.09 ± 5.76* [9.26–10.92] | **9.35 ± 3.85† [8.52–10.18]** | <0.001 |
| Recall | 0.63 ± 0.21*  [0.60–0.67] | 0.47 ± 0.22*  [0.44–0.51] | 0.65 ± 0.23* [0.61–0.68] | 0.47 ± 0.22* [0.43–0.50] | 0.27 ± 0.21*  [0.24–0.31] | 0.27 ± 0.21*  [0.24–0.31] | 0.27 ± 0.23*  [0.24–0.30] | 0.22 ± 0.21*  [0.19–0.26] | 0.31 ± 0.24*  [0.28–0.34] | 0.25 ± 0.21*  [0.22–0.29] | **0.73 ± 0.24†**  **[0.69–0.76]** | <0.001 |
| Precision | 0.28 ± 0.18*  [0.25–0.31] | 0.38 ± 0.22*  [0.35–0.41] | 0.15 ± 0.12* [0.12–0.18] | 0.23 ± 0.16* [0.20–0.27] | 0.11 ± 0.08*  [0.08–0.14] | 0.11 ± 0.09*  [0.08–0.14] | 0.25 ± 0.21*  [0.22–0.28] | 0.27 ± 0.23*  [0.25–0.30] | 0.31 ± 0.23*  [0.28–0.34] | 0.34 ± 0.24*  [0.31–0.37] | **0.46 ± 0.22†**  **[0.43–0.49]** | <0.001 |
| F1 score | 0.35 ± 0.18*  [0.32–0.37] | 0.37 ± 0.17*  [0.34–0.39] | 0.22 ± 0.14* [0.19–0.25] | 0.28 ± 0.16* [0.25–0.31] | 0.13 ± 0.07*  [0.10–0.15] | 0.13 ± 0.08*  [0.10–0.15] | 0.22 ± 0.16*  [0.19–0.24] | 0.21 ± 0.16*  [0.18–0.23] | 0.26 ± 0.17*  [0.24–0.29] | 0.25 ± 0.16*  [0.22–0.27] | **0.53 ± 0.21† [0.50–0.55]** | <0.001 |
| LOGAVD | 0.29 ± 0.24  [0.23–0.34] | 0.55 ± 0.39*  [0.50–0.60] | 0.33 ± 0.27 [0.26–0.38] | 0.54 ± 0.41* [0.48–0.59] | 0.47 ± 0.34*  [0.42–0.52] | 0.46 ± 0.34*  [0.41–0.52] | 0.35 ± 0.30  [0.30–0.40] | 0.32 ± 0.28  [0.27–0.38] | 0.40 ± 0.42*  [0.34–0.45] | 0.41 ± 0.42*  [0.35–0.46] | **0.27 ± 0.22†**  **[0.21–0.32]** | <0.001 |
| ICC (A, 1) |  |  |  |  |  |  |  |  |  |  |  |  |
| Coefficient | 0.91  [0.88–0.94] | 0.73  [0.03–0.90] | 0.88  [0.83–0.91] | 0.73  [0.09–0.89] | 0.75  [0.67–0.81] | 0.75  [0.67–0.81] | 0.88  [0.84–0.91] | 0.90  [0.86–0.93] | 0.81  [0.75–0.86] | 0.80  [0.72–0.86] | **0.93†**  **[0.85–0.96]** |  |
| p-value | < 0.001 | < 0.001 | < 0.001 | < 0.001 | < 0.001 | < 0.001 | < 0.001 | < 0.001 | < 0.001 | < 0.001 | < 0.001 |  |

**Supplementary Table 7: Dice score and LOGAVD of automated white matter lesion segmentation approaches according to lobes in the control group.**

Values in the table are mean ± standard deviation [95% confidence interval] from linear mixed-effects models. Numbers in parentheses indicate the thresholds used in the automated algorithms. The dagger indicates the best-performing algorithm. The asterisks on the remaining algorithms denote a significant difference from the best algorithm. The best performing approach for each metric is represented in bold.

|  | BIANCA  -LOO  (0.9) | BIANCA  - LOO  (0.99) | FreeSurfer | FreeSurfer  -FAST | LPA  (0.4) | LPA  (0.5) | LPA  -FAST  (0.4) | LPA  -FAST  (0.5) | U-Net | p-value |
| --- | --- | --- | --- | --- | --- | --- | --- | --- | --- | --- |
| **Frontal** |  |  |  |  |  |  |  |  |  |  |
| Dice | 0.24 ± 0.20* [0.19–0.27] | 0.11 ± 0.11* [0.05–0.14] | 0.05 ± 0.10* [0.01–0.09] | 0.06 ± 0.10* [0.02–0.10] | 0.13 ± 0.19* [0.08–0.16] | 0.11 ± 0.17* [0.06–0.14] | 0.13 ± 0.17* [0.08–0.16] | 0.10 ± 0.15* [0.04–0.12] | **0.40 ± 0.25† [0.35–0.44]** | <0.001 |
| LOGAVD | 0.17 ± 0.24 [0.08–0.25] | 0.24 ± 0.35* [0.15–0.33] | 0.25 ± 0.37* [0.16–0.34] | 0.28 ± 0.44* [0.19–0.37] | 0.20 ± 0.31 [0.12–0.29] | 0.21 ± 0.32* [0.12–0.29] | 0.26 ± 0.42* [0.17–0.35] | 0.28 ± 0.43* [0.19–0.37] | **0.10 ± 0.17† [0.01–0.19]** | <0.001 |
| **Temporal** |  |  |  |  |  |  |  |  |  |  |
| Dice | 0.07 ± 0.14* [-0.01–0.08] | 0.03 ± 0.06* [-0.07–0.03] | 0.02 ± 0.08* [-0.02–0.05] | 0.02 ± 0.09* [-0.01–0.05] | 0.07 ± 0.17* [0.02–0.10] | 0.07 ± 0.15* [0.00–0.09] | 0.07 ± 0.17* [0.02–0.10] | 0.06 ± 0.15* [-0.00–0.08] | **0.18 ± 0.25† [0.12–0.21]** | <0.001 |
| LOGAVD | 0.03 ± 0.08 [-0.02–0.08] | 0.03 ± 0.09 [-0.01–0.08] | 0.09 ± 0.29 [0.05–0.14] | 0.13 ± 0.40* [0.09–0.18] | 0.03 ± 0.07 [-0.02–0.08] | 0.03 ± 0.06 [-0.02–0.07] | 0.05 ± 0.16 [0.00–0.09] | 0.04 ± 0.14 [-0.00–0.09] | **0.02 ± 0.06† [-0.02–0.07]** | 0.002 |
| **Parietal** |  |  |  |  |  |  |  |  |  |  |
| Dice | 0.18 ± 0.21* [0.12–0.22] | 0.09 ± 0.14* [0.01–0.11] | 0.07 ± 0.15* [0.03–0.12] | 0.06 ± 0.13* [0.02–0.11] | 0.13 ± 0.19* [0.06–0.16] | 0.13 ± 0.20* [0.05–0.15] | 0.15 ± 0.20* [0.07–0.17] | 0.14 ± 0.20* [0.06–0.16] | **0.31 ± 0.26† [0.25–0.35]** | <0.001 |
| LOGAVD | 0.13 ± 0.21 [0.06–0.19] | 0.17 ± 0.31 [0.10–0.23] | 0.19 ± 0.30* [0.12–0.25] | 0.22 ± 0.36* [0.16–0.29] | 0.17 ± 0.25* [0.10–0.24] | 0.15 ± 0.23 [0.08–0.21] | 0.15 ± 0.30 [0.08–0.22] | 0.15 ± 0.31 [0.08–0.22] | **0.06 ± 0.10 † [-0.00–0.13]** | <0.001 |
| **Occipital** |  |  |  |  |  |  |  |  |  |  |
| Dice | 0.09 ± 0.14* [0.03–0.12] | 0.01 ± 0.03* [-0.07–0.02] | 0.03 ± 0.07* [-0.02–0.07] | 0.03 ± 0.07* [-0.02–0.07] | 0.15 ± 0.22 [0.10–0.19] | 0.14 ± 0.21 [0.09–0.17] | 0.17 ± 0.23 [0.12–0.21] | **0.18 ± 0.23† [0.13–0.21]** | 0.16 ± 0.20 [0.11–0.20] | <0.001 |
| LOGAVD | **0.05 ± 0.08† [-0.00–0.11]** | 0.06 ± 0.11 [0.00–0.11] | 0.18 ± 0.32* [0.12–0.23] | 0.18 ± 0.35* [0.13–0.24] | 0.18 ± 0.29* [0.12–0.23] | 0.13 ± 0.22 [0.07–0.18] | 0.14 ± 0.16 [0.08–0.19] | 0.09 ± 0.12 [0.04–0.15] | 0.18 ± 0.17* [0.12–0.23] | <0.001 |
| **Cingulate** |  |  |  |  |  |  |  |  |  |  |
| Dice | 0.19 ± 0.24 [0.11–0.21] | 0.10 ± 0.16* [0.01–0.11] | 0.05 ± 0.14* [0.00–0.09] | 0.05 ± 0.14* [0.00–0.09] | 0.13 ± 0.23* [0.07–0.16] | 0.13 ± 0.22* [0.06–0.16] | 0.09 ± 0.16* [0.03–0.13] | 0.08 ± 0.14* [0.01–0.11] | **0.24 ± 0.27† [0.16–0.26]** | <0.001 |
| LOGAVD | 0.03 ± 0.07 [0.00–0.05] | 0.03 ± 0.10 [0.01–0.06] | 0.08 ± 0.09* [0.06–0.10] | 0.09 ± 0.10* [0.07–0.11] | 0.05 ± 0.08 [0.03–0.07] | 0.04 ± 0.07 [0.02–0.06] | 0.05 ± 0.13* [0.03–0.07] | 0.05 ± 0.12 [0.02–0.07] | **0.02 ± 0.03† [-0.01–0.04]** | <0.001 |
| **Insular** |  |  |  |  |  |  |  |  |  |  |
| Dice | 0.20 ± 0.26 [0.12–0.25] | 0.12 ± 0.18* [0.03–0.16] | 0.07 ± 0.14* [-0.01–0.11] | 0.08 ± 0.16* [-0.01–0.12] | 0.12 ± 0.19* [0.02–0.15] | 0.10 ± 0.19* [-0.01–0.13] | 0.12 ± 0.20* [0.02–0.15] | 0.08 ± 0.14* [-0.04–0.10] | **0.25 ± 0.30† [0.16–0.29]** | <0.001 |
| LOGAVD | 0.03 ± 0.07 [0.00–0.06] | 0.04 ± 0.10 [0.01–0.06] | 0.05 ± 0.16 [0.03–0.08] | 0.07 ± 0.20* [0.05–0.10] | 0.03 ± 0.07 [0.00–0.06] | 0.03 ± 0.08 [0.00–0.06] | 0.04 ± 0.11 [0.01–0.07] | 0.04 ± 0.12 [0.02–0.07] | **0.01 ± 0.04† [-0.01–0.04]** | 0.006 |
| **DWM** |  |  |  |  |  |  |  |  |  |  |
| Dice | 0.41 ± 0.20 [0.36–0.46] | 0.28 ± 0.16* [0.23–0.34] | 0.21 ± 0.20* [0.16–0.26] | 0.21 ± 0.20* [0.16–0.27] | 0.34 ± 0.24* [0.28–0.39] | 0.32 ± 0.25* [0.27–0.37] | 0.36 ± 0.22* [0.30–0.41] | 0.33 ± 0.22* [0.28–0.39] | **0.43 ± 0.23† [0.38–0.48]** | <0.001 |
| LOGAVD | 0.18 ± 0.14 [0.12–0.24] | 0.28 ± 0.29 [0.22–0.34] | 0.37 ± 0.24* [0.31–0.43] | 0.44 ± 0.28* [0.38–0.50] | 0.24 ± 0.24 [0.18–0.29] | 0.21 ± 0.20 [0.15–0.27] | 0.24 ± 0.25 [0.18–0.30] | 0.24 ± 0.27 [0.18–0.30] | **0.17 ± 0.24† [0.11–0.23]** | <0.001 |

**Supplementary Table 8: Validation of white matter lesion volumes measured by automated approaches using age of participants in the Parkinson’s disease group.**

Values are the results of bivariate correlation analysis. The asterisk indicates the highest value.

|  | Pearson correlation coefficient | p-value |
| --- | --- | --- |
| Manual segmentation | 0.434 | < 0.001 |
| BIANCA-LOO (0.9) | 0.477 | < 0.001 |
| BIANCA-LOO (0.99) | 0.442 | < 0.001 |
| BIANCA-training (0.9) | 0.486 | < 0.001 |
| BIANCA-training (0.99) | 0.437 | < 0.001 |
| FreeSurfer | 0.409 | < 0.001 |
| FreeSurfer-FAST | 0.371 | < 0.001 |
| LPA (0.4) | 0.487 | < 0.001 |
| LPA (0.5) | 0.489* | < 0.001 |
| LPA-FAST (0.4) | 0.447 | < 0.001 |
| LPA-FAST (0.5) | 0.445 | < 0.001 |
| U-Net | 0.422 | < 0.001 |

**Supplementary Table 9: Validation of white matter lesion volumes measured by automated approaches using Fazekas and Wahlund scores in the Parkinson’s disease group.**

Values are the results of partial correlation analysis after adjusting for age. The asterisk indicates the highest value. The best performing automated approach is represented in bold.

|  | Fazekas | | Wahlund | |
| --- | --- | --- | --- | --- |
|  | Partial correlation coefficient | p-value | Partial correlation coefficient | p-value |
| Manual segmentation | 0.863* | < 0.001 | 0.874* | < 0.001 |
| BIANCA-LOO (0.9) | 0.767 | < 0.001 | 0.766 | < 0.001 |
| BIANCA-LOO (0.99) | 0.761 | < 0.001 | 0.771 | < 0.001 |
| BIANCA-training (0.9) | 0.723 | < 0.001 | 0.737 | < 0.001 |
| BIANCA-training (0.99) | 0.728 | < 0.001 | 0.732 | < 0.001 |
| FreeSurfer | 0.484 | < 0.001 | 0.502 | < 0.001 |
| FreeSurfer-FAST | 0.485 | < 0.001 | 0.495 | < 0.001 |
| LPA (0.4) | 0.729 | < 0.001 | 0.704 | < 0.001 |
| LPA (0.5) | 0.745 | < 0.001 | 0.728 | < 0.001 |
| LPA-FAST (0.4) | 0.703 | < 0.001 | 0.675 | < 0.001 |
| LPA-FAST (0.5) | 0.720 | < 0.001 | 0.697 | < 0.001 |
| U-Net | **0.805** | < 0.001 | **0.811** | < 0.001 |

**Supplementary Figure 1: Boxplots of evaluation metrics across the automated approaches in the Parkinson's disease group for medium white matter lesion load.**

Comparison of white matter lesion segmentation algorithm performance in Parkinson's disease patients with medium lesion load (5–15 cm³). Performance of 11 automated segmentation algorithms was evaluated against manual segmentation using six agreement metrics: Dice Similarity Coefficient (higher values indicate better agreement), Hausdorff Distance (lower values indicate better agreement), recall (higher values indicate better agreement), precision (higher values indicate better agreement), F1 score (higher values indicate better agreement), and Log-transformed Absolute Volume Difference (LOGAVD; lower values indicate better agreement). The daggers indicate the best-performing algorithms within each metric. The asterisks indicate statistically significant differences compared to the best performer (*p < 0.05, **p < 0.01, ***p < 0.001).

**Supplementary Figure 2: Post-hoc analysis of performance metrics across the automated approaches in the Parkinson's disease group for medium white matter lesion load.**

This figure presents heatmaps of pairwise post-hoc comparisons of automated white matter lesion segmentation algorithms in Parkinson's disease patients with medium lesion load (5–15 cm³). Each cell displays the statistical significance (p-value) of differences in segmentation performance between two algorithms based on six evaluation metrics: Dice similarity coefficient, Hausdorff distance, recall, precision, F1 score, and log-transformed absolute volume difference (LOGAVD). Red indicates greater statistical difference, and white indicates non-significant difference.

**Supplementary Figure 3: Boxplots of evaluation metrics across the automated approaches in the Parkinson's disease group for high white matter lesion load.**

Comparison of white matter lesion segmentation algorithm performance in Parkinson's disease patients with high lesion load (>15 cm³). Performance of 11 automated segmentation algorithms was evaluated against manual segmentation using six agreement metrics: Dice Similarity Coefficient (higher values indicate better agreement), Hausdorff Distance (lower values indicate better agreement), recall (higher values indicate better agreement), precision (higher values indicate better agreement), F1 score (higher values indicate better agreement), and Log-transformed Absolute Volume Difference (LOGAVD; lower values indicate better agreement). The daggers indicate the best-performing algorithms within each metric. The asterisks indicate statistically significant differences compared to the best performer (*p < 0.05, **p < 0.01, ***p < 0.001).

**Supplementary Figure 4: Post-hoc analysis of performance metrics across the automated approaches in the Parkinson's disease group for high white matter lesion load.**

This figure presents heatmaps of pairwise post-hoc comparisons of automated white matter lesion segmentation algorithms in Parkinson's disease patients with high lesion load (>15 cm³). Each cell displays the statistical significance (p-value) of differences in segmentation performance between two algorithms based on six evaluation metrics: Dice similarity coefficient, Hausdorff distance, recall, precision, F1 score, and log-transformed absolute volume difference (LOGAVD). Red indicates greater statistical difference, and white indicates non-significant difference.
